# STEM learning communities promote friendships but risk academic segmentation

**DOI:** 10.1038/s41598-022-15575-y

**Published:** 2022-07-20

**Authors:** Wesley Jeffrey, David R. Schaefer, Di Xu, Peter McPartlan, Sabrina Solanki

**Affiliations:** 1 Department of Sociology, University of California-Irvine, 4215 Social Science Plaza B, Irvine, CA 92617 USA; 2 Department of Education, University of California-Irvine, Irvine, CA 92617 USA; 3 Division of Teaching Excellence and Innovation, University of California-Irvine, Irvine, CA 92617 USA

**Keywords:** Applied mathematics, Human behaviour, Population dynamics, Ecological networks

## Abstract

Universities are increasingly using learning communities (LCs) to promote the academic and social integration of entering students, especially within STEM majors. Examining the causal effect of LCs on student networks is necessary to understand the nature and scope of their impact. This study combines a regression discontinuity design with social network analysis to estimate the effect of a simple LC design on the *size*, *strength*, *structure*, and *composition* of friendship networks among students within the same biological sciences freshman cohort. Results of the quasi-experimental analysis indicate that LC participants acquired one additional friend in the major and increased their share of friends in the LC by 54 percentage-points. Exponential random-graph models that test mediation and alternative friendship mechanisms provide support for the theoretical argument that the LC promoted friendship development by structuring opportunities for interaction through block-registration into courses. Thus, this study shows that even simple LCs can shape the development of friendships through relatively low-cost administrative means. The increased access to resources and support facilitated by the LC is likely beneficial for participating students. However, there is a potential downside when eligibility for participation is determined using academic metrics that separate the student population into distinct classroom environments.

## Introduction

STEM higher education suffers from issues of attrition and academic disparities that threaten to undermine the adequate supply of skilled workers to keep up with societal demand^1^. Nearly half of bachelor’s degree-seeking students leave STEM fields^2^, and those who persist and perform best disproportionately come from advantaged backgrounds^3,4^. Finding ways to help all students thrive in STEM environments is a major goal of the science community^5,6^.

One proposed avenue to promote student persistence and academic success is through Learning Communities (LCs)^7–10^. In 2019, an estimated 13% of first-year students and 22% of seniors reported having participated in some form of LC^11^. In general, institutions construct LCs by grouping students through some combination of shared courses, a residential living component, active learning strategies, and informal activity with the goal of promoting student academic and social integration^12–14^. Without the aid of structured interventions such as LCs^15^, students must make connections and find support to navigate the new college and STEM environment largely on their own. Yet, while LCs have been shown to promote performance and persistence in STEM, their direct impact on social integration in terms of student friendships remains unclear^8,10,16–18^.

Indeed, despite intuition regarding how LCs may guide the development of friendships, there is surprisingly little evidence establishing a causal relationship. Correlational studies have linked LC participation to positive relational outcomes, such as increased socializing^19^, heightened exposure and network formation^20^, and social support^21^. However, because universities often make LC participation voluntary, confounds between the types of students who opt into LCs and student outcomes are inevitable, thereby precluding causal inferences^8^. Thus, the effect of LCs on friendship has yet to receive the rigorous causal evaluation needed to demonstrate their promise.

In this study, we extend prior work on college LCs by exploring the causal impact of LCs on friendship networks in a unique setting where students are assigned to participate in a LC using a strict SAT math score cutoff. This enables us to utilize a regression discontinuity design (RDD) that can credibly support causal inferences because assignment to treatment creates a scenario that is “as good as randomized” for individuals proximate to the threshold^22,23^. Accordingly, results from this study advance our understanding of the link between organizational practices and relational outcomes generally, and specifically its importance for helping students build meaningful connections with peers in STEM.

## Background

While social integration is important throughout college, it is particularly critical during the freshman year when students transition into postsecondary schooling^12,24^. Advocates of LCs point out that social integration can improve motivation^25,26^ and provide access to resources and information needed to succeed academically^27,28^. Although social integration has multiple dimensions^29^, encompassing faculty, staff, and peer interactions^12,30^, we focus on the friendship networks that first-year students develop within their major. Friendships are crucial in college^31,32^ as they represent some of the strongest influences on students’ attitudes, values, and behaviors^33^. Within the STEM context, friendships are a key factor promoting persistence^34,35^ and academic success^28,36^.

Evidence from the K-12 context provides reason to suspect that LCs promote friendship by acting as “foci” to structure student interaction patterns^37^. According to focus theory, foci are any “social, psychological, legal, or physical entity around which joint activities are organized” and which “actively bring people together or passively constrain them to interact” (Ref.^38^, pp. 1016, 1018). By enhancing proximity and promoting regular interaction opportunities, foci are a powerful force behind the development of positive sentiments, relationships, and their change throughout the life course^39–41^. Within secondary schools, research has demonstrated the importance of tracking^42^ and clustered sets of courses^43^ for understanding processes of friendship formation among students. Likewise, some work within higher education has also highlighted the association between shared classes^44^ and majors^45^ and the relationships that arise. Thus, the active manipulation of which students attend class together—at the core of the LC model—will likely shape which friendships emerge.

Based upon insight from focus theory, we expect the LC to concentrate friendships among students assigned to the same courses. We also expect the community cultivated by the LC to lead to more friendships and friendships that are stronger and more group-based than outside the LC. However, an often-overlooked side effect of these processes is that some potential friendships will be inadvertently discouraged. Students placed in the same classroom are primed for friendship while those placed in different classrooms face a structural barrier^42,46,47^. Hence, the LC may create divisions within the student body, which can exacerbate inequality^48,49^. Our analysis considers multiple friendship network outcomes—size, strength, structure, and composition (see “Methods” section for details)—as a way to evaluate the intended goal of social integration, while being cognizant of such unintended consequences^19^.

Our results indicate that participating in the LC led to an additional friend in the major, although this effect was only marginally significant. In addition, LC participation led to a 54 percentage-point increase in students’ share of friends in the LC. We did not find evidence that participating in the LC altered the strength or structure of students’ friendship networks. Follow-up mediation analyses substantiate the theoretical expectation that increased opportunity for interaction brought about through the LC’s block-registration into classes is the main mechanism responsible for the observed differences in friendship network outcomes.

## Methods

### Data and setting

Data come from two sequential cohorts of first-time entering biological sciences freshmen at a large, selective, public R1 university in the Western United States. The case under study represents a diverse environment in terms of race/ethnicity, socioeconomic background, and gender. Namely, the major cohort across years is predominantly female, with around half of students considered first-generation, and about 30–40% classified as underrepresented minorities (URM) in terms of racial/ethnic status. During the final week of Fall term, electronic surveys were sent to the entire freshman cohort (LC participants and non-participants) to collect data on friendship ties within the major and various aspects of student background to serve as controls (> 93% response rate). Information on LC participation and additional student demographic data was provided by the university. The study design and procedures were reviewed and approved by the Institutional Review Board of the University of California, Irvine.

### Learning community design

For each cohort, the department implemented a simple LC program by block-registering participating students into the same introductory biology and chemistry courses (see SI Appendix Sect. S1). Eligibility for placement into the program was determined using a strict SAT math cutoff score because prior institutional research had identified this metric as a strong predictor of performance and persistence in the major. Each year the freshman cohort consisted of around 1000 students, and approximately 300 students (or one-third) below the cutoff were assigned to participate in the LC. Along with being placed into the same biology and chemistry courses, all participating students took an additional seminar together that met weekly for one hour. Students were split into groups of about 30 students for these weekly meetings that were designed to promote study skills, career advice, and help with navigating the academic environment. This LC design is relatively easy, low-cost, and the predominant model on large campuses, compared to more extensive LCs utilized in smaller settings^7^.

### Friendship network measures

We draw upon four basic egocentric measures of students’ friendship networks^50^. Network size refers to the number of friends with whom a focal student is connected and is measured using *total degree*, where we do not differentiate who named whom as a friend (the focal student or the peer)^51^. Tie strength reflects the idea that relationships vary along dimensions such as closeness, intensity, and meaningfulness and is measured through the *count of mutual ties*—whereby both students acknowledge the relationship by naming each other as friends^27,52,53^. Network structure recognizes that students not only have friends, but that those friends may be connected to one another. We use *density* as our measure of network structure, calculated as the number of observed ties among a focal student’s friends divided by the number of potential ties^51^. Finally, network composition refers to the characteristics of people in one’s network (e.g., how homogenous one’s friends are). We use the *proportion of friends in the learning community* as our measure of network composition since, given the design of the LC, we expect opportunities and subsequent friendships with LC participants to vary greatly depending upon whether a student belongs to the LC.

### Analytic strategy

Using survey and administrative data from two consecutive first-year cohorts, we tested the LC effect on friendship in two steps. First, we estimate the causal effect of LC participation through a RDD that effectively compares friendship outcomes among students whose SAT math scores placed them just above versus just below the LC threshold. Second, we estimated a series of social network models that test whether the impact of LC participation on friendship was mediated by LC organizational factors, versus alternative mechanisms that may have coincided with the assignment of students to courses and sections (i.e., potential confounds). This second step replicates the findings of the RD analysis and offers insight to *how* the LC had its observed effects.

### Regression discontinuity approach

The regression discontinuity (RD) approach has been widely used in social science as a compelling quasi-experimental design to estimate program impacts when eligibility to a treatment is determined by whether an individual’s score exceeds a designated threshold or cut-point^23^. This creates a situation that approximates a “local randomization”^22^, where the major premise is that within a specified bandwidth around the cutoff, individuals would not be expected to differ significantly from one another, other than eligibility to program participation. In the case of this study, RD is warranted because the program uses a specific cutoff score to determine each student’s eligibility to participate in the LC; freshmen with SAT math scores below a cutoff of 600 were assigned to participate in the LC. If we assume the underlying relationship between SAT math score and friendship network measures follows a continuous relationship, and nothing other than the LC participation varies discontinuously at the cutoff, then we may attribute any observed discontinuity in friendship network outcomes at the cutoff to LC participation.

To deal with issues of noncompliance where a small proportion of students below the cutoff were exempted from participating in the LC (see SI Appendix Sect. S3), we use a fuzzy RD design. Specifically, we use learning community eligibility as an instrumental variable for actual participation in the first-year program with a two-stage least squares strategy^54^. Namely, we derive estimates of the “local average treatment effects”^55^ (or LATE) through a pooled local polynomial regression within a bandwidth of ± 70 points. For all models measuring the causal impact of the intervention on the four network outcomes, we draw upon the following equations:

1 $$\begin{aligned} {\text{Enroll}}_{{\text{i}}} & = \gamma_{0} + \, \gamma_{{1}} \left( {{\text{Below}}_{{\text{i}}} } \right) + \gamma_{{2}} \left( {{\text{SAT Math Distance}}_{{\text{i}}} } \right) + \gamma_{{3}} ({\text{SAT Math Distance}}_{{\text{i}}} \, *\,\,{\text{SAT}}\,\,{\text{Math Distance}}_{{\text{i}}} ) \, \\ & \quad + \, \gamma_{{4}} \left( {{\text{Below}}_{{\text{i}}} *{\text{ SAT Math Distance}}_{{\text{i}}} } \right) + {\text{X}}_{{\text{i}}} + \mu_{{\text{i}}} \\ \end{aligned}$$

2 $$\begin{aligned} {\text{Y}}_{{\text{i}}} & = \delta_{0} + \delta_{{1}} (\widehat{Enroll}_{{\text{i}}} ) +_{ } \delta_{{2}} \left( {{\text{SAT Math Distance}}_{{\text{i}}} } \right) + \,\delta_{{3}} ({\text{SAT Math Distance}}_{{\text{i}}} {\text{*SAT Math}}\,\,{\text{Distance}}_{{\text{i}}} ) \\ & \quad + \delta_{{4}} \left( {{\text{Below}}_{{\text{i}}} *{\text{ SAT Math Distance}}_{{\text{i}}} } \right) + {\text{X}}_{{\text{i}}} + \varepsilon_{{\text{i}}} \\ \end{aligned}$$

Equation (1) represents the first stage of the regression, where we predict LC enrollment as a function of eligibility for placement. *Below*_i_ is a binary variable indicating whether the student was assigned to the LC based on SAT math score eligibility; *SAT Math Distance*_*i*_ is the difference between the student’s math SAT score and the cutoff threshold (i.e., 600); *SAT Math Distance*_*i*_ * SAT Math Distance_*i*_ is a quadratic term that allows for nonlinear relationships between the running variable and the outcome; *Below*_*i*_ * SAT Math Distance_*i*_ is an interaction term that allows different slopes above and below the threshold; *X*_*i*_ is a vector of individual-level covariates as outlined above. Equation (2) represents the second stage of the regression, where we use the predicted probability of enrollment to estimate the local average treatment effect as indicated by the δ_1_ coefficient. We estimate the impact of the LC on each network outcome separately using the *ivregress* command in STATA version 16.1 (https://www.stata.com).

### Social network analysis

We used an ERGM^56^ to estimate the factors that promoted friendships between students at the end of their first quarter on campus. The ERGM considers all possible directed dyads among the sample of students, where an *i* → *j* friendship was modeled separately from a *j* → *i* friendship. The model estimates the probability of observing a given network conditioned on the set of effects present in the model. We use two types of effects: nodal covariates represent student characteristics (e.g., LC participation, gender) and dyadic covariates represent similarity (i.e., homophily) or co-presence of students (e.g., in the LC, classes). Specific effects are listed in SI Appendix Sect. S4. Estimated coefficients are interpretable as the log-odds of observing a friendship in a given dyad conditional on the rest of the network. For a given effect, exponentiating the estimated coefficient indicates how a one-unit change affects the odds of a tie, assuming all other model effects remain constant. We estimated a separate ERGM for each first-year student cohort using the *statnet* package in R version 4.1.0 (https://www.r-project.org)^57^.

### Research ethics

The study design and procedures were reviewed and approved by the Institutional Review Board. All research was performed in accordance with relevant guidelines and regulations. The need of informed consent was waived by the Institutional Review Board of the University of California, Irvine, due to registration under exemption category 1.

## Results

### Descriptive evidence

**Figure 1:**
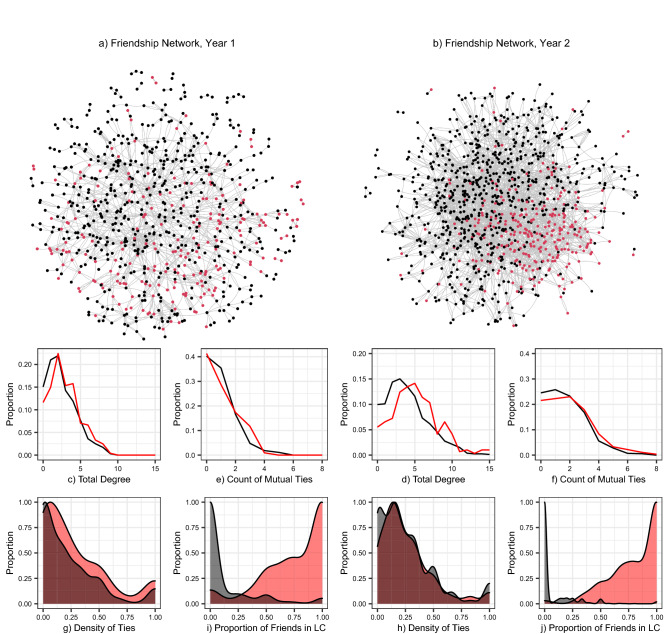
The biological sciences friendship networks and distributions of network outcomes by year. (**a**,**b**) Omit isolates and highlight segmentation of LC from non-LC students, which is stronger in Year 2, likely due to programmatic changes (see SI Appendix Sect. S1). The Year 2 network is also more densely connected, with an average outdegree of 2.93, vs 1.72 the prior year, which we attribute to differences in the survey instrument (see SI Appendix Sect. S1). (**c**–**j**) reflect the distribution of each of our network outcomes in each year. Node, line, and density plot colors indicate LC students (red) and non-LC students (black) in each panel.

Figure 1 presents the friendship networks and distributions of network outcomes for the full set of first-year students (see “Methods” section for details). Descriptively, we find that LC students were more socially integrated, with significantly more friends and a greater share of friends in the LC compared to non-participants across years (panels c,d,i,j), but more mutual ties (panels e–f) and more dense networks (panels g,h) in only one of the years (see SI Appendix Fig. S1). Additionally, in examining the odds of having *no friends* (i.e., being an “isolate”) in the major, LC participants were 50% less likely to be an isolate, compared to non-participants (p < 0.01; SI Appendix Fig. S2). The sociograms in panels *a-b* make clear the network segmentation based on LC status, which is stronger in Year 2 (see SI Appendix Sect. S1).

### Impact of LC on friendship network outcomes

**Figure 2:**
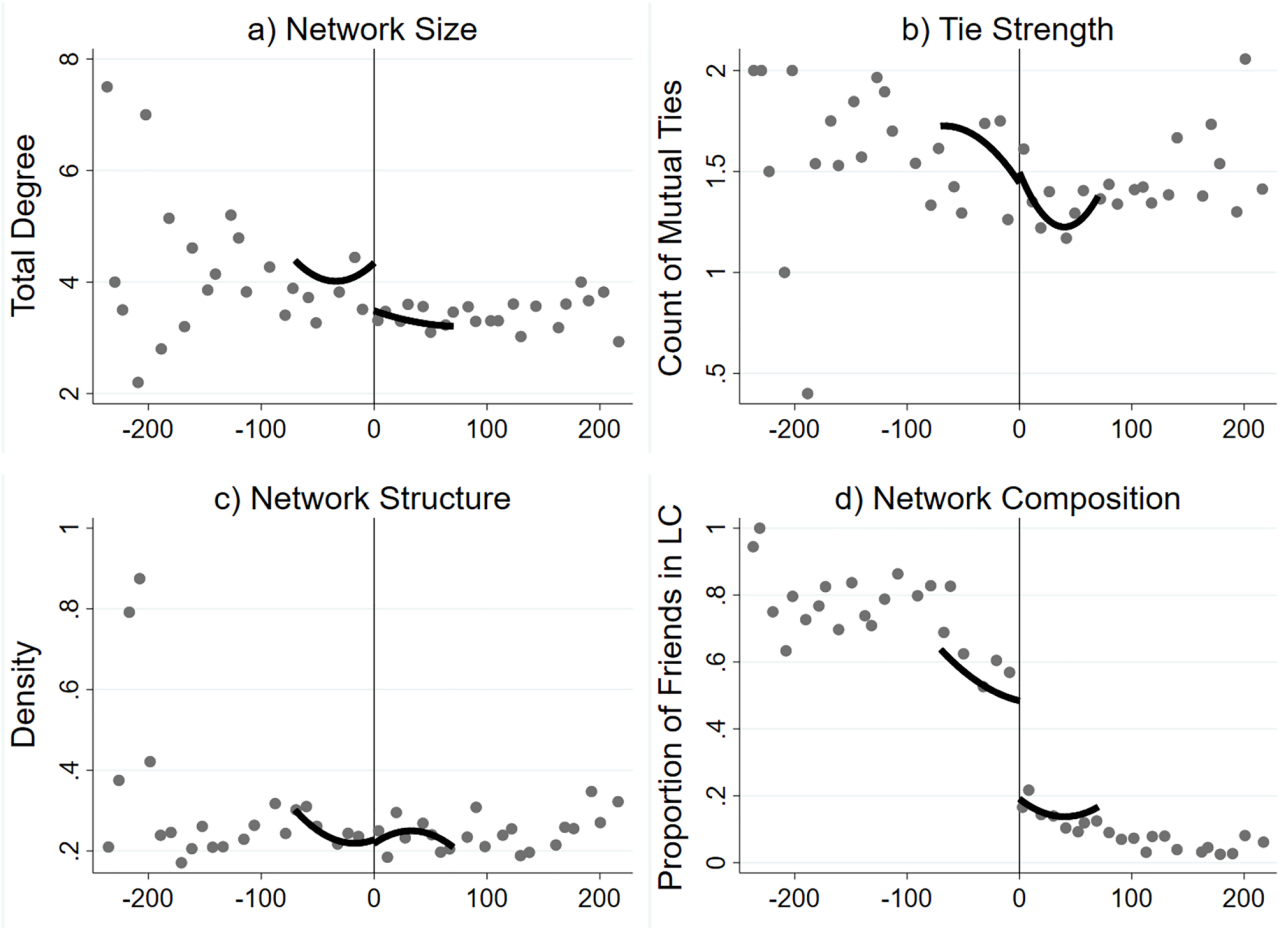
RD plots of friendship network outcomes at SAT math score cutoff. In (**a**–**d**), RD plots are generated on the pooled sample across years, using the *rdplot* command in STATA with bin size selected using the default esmv method (see Ref.^58^ for details). While average outcomes within each bin are plotted on the entire sample, predicted quadratic lines are only fitted to those within ± 70 points around the eligibility threshold. Fitted lines adjust for mass points in the data and control for the following: race/ethnicity, gender, first-generation student status, low-income status, high school GPA, Fall term cumulative GPA, survey completion status, and year.

While the descriptive statistics presented above provide support for the positive association between LC participation and friendship development, it is unclear whether observed differences are due to the impact of the LC or baseline differences between LC participants and non-participants. Figure 2 visualizes the discontinuity of the four network outcomes at the SAT cutoff, where quadratic prediction lines are fitted within a bandwidth of ± 70 points around the threshold. Overall, we find visual evidence for a discontinuity in network size and network composition at the cutoff, but no discontinuity in tie strength or network structure. These patterns are supported by statistical estimates of the local average treatment effect (LATE) based on pooled local polynomial regressions (see SI Appendix Fig. S3): LC participation led to an additional friend in the major (p < 0.10) as well as a 54 percentage-point increase, on average, in the share of friends in the first-year program (p < 0.001). No significant effects were observed for the count of mutual ties (p > 0.10) or network density (p > 0.10).

To aid interpretation of our RD results, we use the LATE estimates to calculate predicted network outcomes for LC participants compared to non-LC participants. As shown in SI Appendix Fig. S4, LC participants are expected to average 4.25 fellow first-year majors as friends compared to 3.25 for their non-LC counterparts. In addition, the LC affected whom students befriend: LC participants are predicted to have almost 70% of their friends in the LC, whereas their similar non-LC peers are predicted to have less than 20% of their friends in the LC program. Together, these results demonstrate that the LC had friendship network *size* and *segmentation effects* for students around the cutoff.

Given we found a marginally significant effect of LC participation on network size, in the next section, we specifically test the hypothesized mechanism through which the LC shaped friendship volume: namely, heightened opportunity to interact brought about through block-registration. Because the RD approach assumes that no other meaningful differences exist that could explain the gap at the cutoff, the follow-up network analyses provide additional validation by explicitly modeling alternative explanations that could plausibly lead to the differences we observe.

### Network mediation analysis

Having demonstrated the effect of the LC on student friendships, we turn to testing the proposed mechanism by which the LC operated. This mediation analysis uses the full network of students each year and an exponential random-graph model, or ERGM^56^. Parameter estimates reflect the likelihood that a friendship will be present, versus absent, in a given dyad based on a given effect. Marginal effects are used to test for mediation^59^.

**Figure 3:**
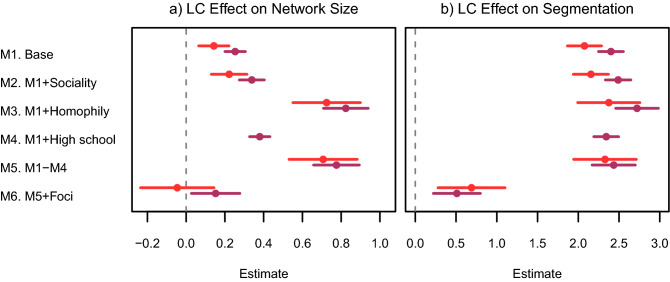
ERGM estimates testing alternative explanations and mediation of LC on network size and segmentation. Model 1 is the base model and demonstrates the main LC effect. Models 2–4 introduce measures of sociality, homophily, and same high school separately. Model 5 includes all alternative explanations simultaneously. Model 6 introduces the foci variables while controlling for all alternative mechanisms. (**a**,**b**) Provide the coefficients from the specified ERGMs for network size and segmentation, respectively (see SI Appendix Sect. S4 for details). Plotted distance from zero corresponds to the direct magnitude of the LC effect on each network outcome across models. Point estimates and 95% confidence intervals correspond to Year 1 (light red) and Year 2 (dark red) in both panels. Full model results in SI Appendix Figs. S16 and S17.

ERGM findings mirror the causal analysis. LC participants had significantly more friends overall, and significantly more friends in the LC than non-participants. As shown for the Base model in Fig. 3 (M1), the odds of a friendship were 1.1–1.3 times greater for LC participants vs. non-participants (panel *a*) and LC participants were 8–11 times more likely than non-participants to be friends with LC students (panel *b*).

While we hypothesize that these effects are driven by the LC serving as a foci for friendship activity, other possibilities exist. (1) The LC may have concentrated more sociable groups of students who would otherwise make more friends even in the absence of the LC program^28^. (2) The LC may have drawn students who shared greater similarities than students outside the LC and hence would be more likely to become friends even in the absence of the LC program. Coupled with the power of homophily in driving friendship^60^, this could have created a more fertile friendship environment within the LC. (3) LC participants may have been more likely than non-participants to know one another before entering the LC program.

Models 2–5 test for these possibilities and show that they largely affected friendship in the expected manner (see SI Appendix Sect.  S4). In at least one of the years, first-generation, low-income, and commuter students named fewer friends, while higher GPA and female students named more friends, net of LC participation. Students were also likely to befriend peers who were similar in race/ethnicity, first-generation status, high-school GPA, and gender, as well as retain friends from high school. However, none of these alternative explanations accounted for the observed effect of LC participation on friendship (AME results described in SI Appendix Sect. S4 support this inference).

Model 6 introduces effects to account for the assignment of LC students to specific classes and sections. These are powerful forces driving friend selection: students were approximately 1.5–4 times more likely to befriend a classmate, and LC students were 8.5–12 times more likely to befriend someone in their same section (see SI Appendix Sect. S4). As shown in Fig. 3, with the introduction of foci effects in M6, the positive estimates of LC participation on network size (panel a) and segmentation (panel b) disappeared. The marginal effect estimates support this inference and indicate that foci mediated all of the effect of the LC on both network size and segmentation, revealing a suppression effect. Combined, these findings support our theoretical argument that the LC served as a foci that provided opportunities and support for friendship development.

## Discussion

In this study, we combined a quasi-experimental approach with social network analysis to understand how learning communities shape friendships within a STEM major. Based on the regression discontinuity design, our analysis offers evidence that the LC led to an additional friend in the major, although the effect was only marginally significant. In addition, we found that LC participation significantly and substantially increased the segmentation of student friendship networks. By contrast, students developed equally close and group-based friendships regardless of LC participation. The latter may be a product of the first-year environment, where it is paramount for students to rebuild their networks and develop sources of companionship and support. Such a drive may be so fundamental^61^ that it can be met regardless of the added interaction opportunities provided by the LC.

These findings have important policy implications for efforts to facilitate connections among college students^31,32^, specifically within STEM majors^28,35^. Network science demonstrates how the interplay of friend selection^62^ and influence processes^36,63^ contribute to academic performance differentials in schools^28^, potentially exacerbating gaps among students who enter college at varying achievement levels^48^. College administrators should be attentive to these dynamics when designing curricular interventions^64,65^. Namely, the size and segmentation effects found here could have both intended and unintended academic consequences^19^.

On one hand, students are likely to benefit from the additional friend in the major^27^ that the simple LC helped to promote. Friends provide important help and support with the adjustment during the transition to college^12,13,31^. Particularly in competitive STEM majors, friends can be valuable sources of social capital by improving access to academic resources^27,36^ and fostering a heightened sense of belonging^66^. As a result, LCs offer to improve persistence and success in STEM through their impact on the social integration of entering students^10,28,35^.

On the other hand, by using SAT math score as the eligibility criterion, the LC promoted some friendships at the expense of others. Namely, through block-scheduling of coursework, the LC effectively sorted friendships by prior performance, making relationships between higher- and lower-performing students less likely^42,46^. Thus, findings from this study have significance for discussions around curricular interventions that actively sort individuals into peer groups, such as remedial education, “ability” grouping, or tracking, by revealing how such interventions may affect patterns of relational ties. Because friendships represent a unique influence on the lives of students^27,31,33^—distinct from roommates^67^ and larger classroom or peer groups^68–70^—the structuring of friendships could magnify STEM academic disparities^71^ by inhibiting diverse networks inside and outside the classroom^72–74^.

Our analysis is not without limitations. Although our quasi-experimental approach represents a more rigorous investigation relative to past work, the estimated effect is local and only applicable to individuals around the threshold. Future work may wish to extend our analysis by conducting randomized controlled trials that would enable us to estimate the average treatment effects of the LC. In a similar vein, our in-depth analysis relies upon data from one STEM major and at one location. Future research would benefit from focusing on a broader set of majors and across institutional types. Finally, whereas we utilized mutuality as one measure of tie strength, we acknowledge that there may be other ways to capture this construct that future work could explore^75,76^.

Notwithstanding these limitations, the present study makes important theoretical and empirical contributions regarding the role of foci in structuring networks. First, our results reveal that by constructing foci, network interventions may have both intended and unintended consequences for group outcomes^65,77^. Thus, for higher education administrators, manipulating coursework is a powerful form of network engineering that requires attentiveness to potential social as well as academic consequences. Second, we show that even foci with relatively low levels of constraint on interpersonal interaction can shape social relationships in significant ways^38^. While the current LC design did not impact mutuality or friendship density, future interventions may be able to impact these outcomes by focusing interactions more intensely. Such efforts could include placing students into study partnerships or groups that are even smaller than the classrooms and ~ 30-person study sections in the observed LC. Such actions could be especially fruitful for fostering network connections and supporting social integration for students from diverse backgrounds and other groups historically at greater risk of STEM attrition.

## Supplementary Information

Supplementary Information.

## Data Availability

The datasets generated and/or analyzed during the current study are not publicly available due to the risk for deductive disclosure but are available from the corresponding author upon reasonable request.

## References

[1] Xie Y, Fang M, Shauman K (2015). STEM education. Annu. Rev. Sociol..

[2] Chen, X. STEM attrition: College students' paths into and out of STEM fields. *National Center for Education Statistics*. Retrieved from http://ies.ed.gov/pubsearch/pubsinfo.asp?pubid=2014001rev. Accessed 22 September 2021.

[3] Huang G, Taddese N, Walter E (2000) Entry and persistence of women and minorities in college science and engineering education. *National Center for Education Statistics*. Retrieved from https://eric.ed.gov/?id=ED566411. Accessed 22 September 2021.

[4] Hurtado, S., Eagan, K., & Chang, M. *Degrees of Success: Bachelor’s Degree Completion Rates among Initial STEM Majors* (Higher Education Research Institute, Los Angeles, CA) (2010).

[5] National Science Foundation, Broadening Participation Working Group (2014) Pathways to broadening participation in response to the CEOSE 2011–2012 recommendation. *National Science Foundation*. Retrieved from https://www.nsf.gov/pubs/2015/nsf15037/nsf15037.pdf. Accessed 22 Sep 2021.

[6] James SM, Singer SR (2016). From the NSF: The National Science Foundation’s investments in broadening participation in science, technology, engineering, and mathematics education through research and capacity building. CBE Life Sci. Educ..

[7] Smith BL, MacGregor J, Matthews R, Gabelnick F (2004). Learning communities: Reforming undergraduate education.

[8] Andrade MS (2007). Learning communities: Examining positive outcomes. J. Coll. Stud. Ret..

[9] Maton KI, Pollard SA, McDougall Weise TV, Hrabowski FA (2012). Meyerhoff Scholars Program: A strengths-based, institution-wide approach to increasing diversity in science, technology, engineering, and mathematics. Mt Sinai J. Med..

[10] Dagley M, Georgiopoulos M, Reece A, Young C (2016). Increasing retention and graduation rates through a STEM learning community. J. Coll. Stud. Ret..

[11] National Survey of Student Engagement (2015) *Engagement Insights: Survey Findings on the Quality of Undergraduate Education—Annual Results 2015* (Bloomington, IN).

[12] Tinto V (1987). Leaving college: Rethinking the causes and cures of student attrition.

[13] Tinto V (2003). Learning better together: The impact of learning communities on student success. Higher Educ. Monogr. Ser..

[14] Otto S, Evins MA, Boyer-Pennington M, Brinthaupt TM (2015). Learning communities in higher education: Best practices. Journal of Student Success and Retention.

[15] Boda Z, Elmer T, Vörös A, Stadtfeld C (2020). Short-term and long-term effects of a social network intervention on friendships among university students. Sci. Rep..

[16] Hotchkiss JL, Moore RE, Pitts MM (2006). Freshman learning communities, college performance, and retention. Educ. Econ..

[17] Whalen DF, Shelley MC (2010). Academic success for STEM and non-STEM majors. J. STEM Educ..

[18] Xu D, Solanki S, McPartlan P, Sato B (2018). EASEing students into college: The impact of multidimensional support for underprepared students. Educ. Res..

[19] Jaffee D, Carle A, Phillips R, Paltoo L (2008). Intended and unintended consequences of first-year learning communities: An initial investigation. J. First-Year Exp. Stud. Trans..

[20] Tinto V, Goodsell A (1994). Freshman interest groups and the first-year experience: Constructing student communities in a large university. J. First Year Exp. Stud. Trans..

[21] Domizi D (2008). Student perceptions about their informal learning experiences in a first-year residential learning community. J. First Year Exp. Stud. Transit..

[22] Lee DS, Lemieux T (2010). Regression discontinuity designs in economics. J. Econ. Lit..

[23] Jacob, R., Zhu, P., Somers, M.A., & Bloom, H. *A Practical Guide to Regression Discontinuity* (MDRC, New York, NY, 2012).

[24] Hays RB, Oxley D (1986). Social network development and functioning during a life transition. J. Pers. Soc. Psychol..

[25] Freeman TM, Anderman LH, Jensen JM (2007). Sense of belonging in college freshmen at the classroom and campus levels. J. Exp. Educ..

[26] Zumbrunn S, McKim C, Buhs E, Hawley LR (2014). Support, belonging, motivation, and engagement in the college classroom: A mixed method study. Instr. Sci..

[27] Hasan S, Bagde S (2013). The mechanics of social capital and academic performance in an Indian college. Am. Sociol. Rev..

[28] Stadtfeld C, Vörös A, Elmer T, Boda Z, Raabe IJ (2019). Integration in emerging social networks explains academic failure and success. Proc. Natl. Acad. Sci. USA.

[29] Kraemer BA (1997). The academic and social integration of Hispanic students into college. Rev. High Educ..

[30] Nora A (1993). Two-year colleges and minority students’ educational aspirations: Help or hindrance. Higher Educ. Handb. Theory Res..

[31] McCabe, J.M. *Connecting in College: How Friendship Networks Matter for Academic and Social Success* (University of Chicago Press, Chicago, IL, 2016).

[32] Felten, P., & Lambert, L. M. *Relationship-rich Education: How Human Connections Drive Success in College* (Johns Hopkins University Press, Baltimore, MD, 2020).

[33] Hallinan MT (1981). The peer influence process. Stud. Educ. Eval..

[34] Thomas SL (2000). Ties that bind: A social network approach to understanding student integration and persistence. J. Higher Educ..

[35] Turetsky KM, Purdie-Greenaway V, Cook JE, Curley JP, Cohen GL (2020). A psychological intervention strengthens students’ peer social networks and promotes persistence in STEM. Sci. Adv..

[36] Dokuka S, Valeeva D, Yudkevich M (2020). How academic achievement spreads: The role of distinct social networks in academic performance diffusion. PLoS ONE.

[37] Epstein JL, Karweit N (1983). Friends in school: Patterns of selection and influence in secondary schools.

[38] Feld SL (1981). The focused organization of social ties. AJS.

[39] Rivera MT, Soderstrom SB, Uzzi B (2010). Dynamics of dyads in social networks: Assortative, relational, and proximity mechanisms. Annu. Rev. Sociol..

[40] Mollenhorst G, Volker B, Flap H (2014). Changes in personal relationships: How social contexts affect the emergence and discontinuation of relationships. Soc. Netw..

[41] Thomas RJ (2019). Sources of friendship and structurally induced homophily across the life course. Sociol Perspect.

[42] Kubitschek WN, Hallinan MT (1998). Tracking and students' friendships. Soc. Psychol. Q.

[43] Frank KA, Muller C, Mueller AS (2013). The embeddedness of adolescent friendship nominations: The formation of social capital in emergent network structures. AJS.

[44] Kossinets G, Watts DJ (2009). Origins of homophily in an evolving social network. AJS.

[45] Wimmer A, Lewis K (2010). Beyond and below racial homophily: ERG models of a friendship network documented on Facebook. AJS.

[46] Hallinan MT, Sørensen AB (1985). Ability grouping and student friendships. Am. Educ. Res. J..

[47] Leszczensky L, Pink S (2015). Ethnic segregation of friendship networks in school: Testing a rational-choice argument of differences in ethnic homophily between classroom-and grade-level networks. Soc. Netw..

[48] DiMaggio P, Garip F (2012). Network effects and social inequality. Annu. Rev. Sociol..

[49] Johnson AM (2019). ‘‘I can turn it on when i need to’’: Pre-college Integration, culture, and peer academic engagement among black and Latino/a engineering Students. Sociol. Educ..

[50] Perry BL, Pescosolido BA, Borgatti SP (2018). Egocentric network analysis: Foundations, methods, and models.

[51] Wasserman S, Faust K (1994). Social network analysis: Methods and applications.

[52] Hartup WW, Stevens N (1997). Friendships and adaptation in the life course. Psychol. Bull..

[53] Vaquera E, Kao G (2008). Do you like me as much as I like you? Friendship reciprocity and its effects on school outcomes among adolescents. Soc. Sci. Res..

[54] Imbens GW, Lemieux T (2008). Regression discontinuity designs: A guide to practice. J. Econom..

[55] Imbens GW, Angrist JD (1994). Identification and estimation of local average treatment effects. Econometrica.

[56] Robins G, Pattison P, Kalish Y, Lusher D (2007). An introduction to exponential random graph (p*) models for social networks. Soc. Netw..

[57] Handcock MS, Hunter DR, Butts CT, Goodreau SM, Morris M (2008). Statnet: Software tools for the representation, visualization, analysis and simulation of network data. J. Stat. Softw..

[58] Calonico S, Cattaneo MD, Titiunik R (2015). Optimal data-driven regression discontinuity plots. J. Am. Stat. Assoc..

[59] Duxbury SW (2021). The problem of scaling in exponential random graph models. Sociol. Methods Res..

[60] McPherson M, Smith-Lovin L, Cook JM (2001). Birds of a feather: Homophily in social networks. Annu. Rev. Sociol..

[61] Kadushin C (2012). Understanding social networks: Theories, concepts, and findings.

[62] Flashman J (2012). Academic achievement and its impact on friend dynamics. Sociol. Educ..

[63] Carrell SE, Sacerdote BI, West JE (2013). From natural variation to optimal policy? The importance of endogenous peer group formation. Econometrica.

[64] Cox AB (2017). Cohorts, ‘‘siblings’,’ and mentors: Organizational structures and the creation of social capital. Sociol. Educ..

[65] Valente TW (2012). Network interventions. Science.

[66] Nunn LM (2021). College belonging: How first-year and first-generation students navigate campus life.

[67] Garlick R (2018). Academic peer effects with different group assignment policies: Residential tracking versus random assignment. Am. Econ. J. Appl. Econ..

[68] Carrell SE, Fullerton RL, West JE (2009). Does your cohort matter? Measuring peer effects in college achievement. J. Labor. Econ..

[69] Lomi A, Snijders TA, Steglich CE, Torló VJ (2011). Why are some more peer than others? Evidence from a longitudinal study of social networks and individual academic performance. Soc. Sci. Res..

[70] Poldin O, Valeeva D, Yudkevich M (2016). Which peers matter: How social ties affect peer-group effects. Res. High Educ..

[71] Raabe IJ, Boda Z, Stadtfeld C (2019). The social pipeline: How friend influence and peer exposure widen the STEM gender gap. Sociol. Educ..

[72] Burt RS (2004). Structural holes and good ideas. AJS.

[73] Oakes J (2005). Keeping track: How schools structure inequality.

[74] Park JJ et al. (2021) Who are you studying with? The role of diverse friendships in STEM and corresponding inequality. *Res. High Educ.*10.1007/s11162-021-09638-8.

[75] Marsden PV, Campbell KE (1984). Measuring tie strength. Soc. Forces.

[76] Mattie H, Engø-Monsen K, Ling R, Onnela JP (2018). Understanding tie strength in social networks using a local “bow tie” framework. Sci. Rep..

[77] Sørensen AB (1970). Organizational differentiation of students and educational opportunity. Sociol. Educ..

