# Supplementary material for: STEM learning communities promote friendships but risk academic segmentation
Source: Sci Rep. 2022 Jul 20;12:12442. doi: 10.1038/s41598-022-15575-y (PMC9300596; doi:10.1038/s41598-022-15575-y)
Supplement: Supplementary file 1 — Supplementary Information. [file 41598_2022_15575_MOESM1_ESM.pdf]

STEM Learning Communities Promote Friendships but Risk Academic Segmentation

Wesley Jeffrey, David R. Schaefer, Di Xu, Peter McPartlan, Sabrina Solanki

SI Appendix

Section 1: Description of LC Program, Data Collection, Sample Characteristics, and Missing Data

Learning Community Design

Figure S5 provides an overview of the learning community program, including eligibility requirements and the following types of program support:

(1) Academic remediation: LC students are required to take an additional developmental chemistry course online the summer prior to college matriculation. This course is designed to prepare potential biological sciences majors for college-level courses in chemistry and biology.

(2) Academic and social support: Students are placed into cohorts where they are matched with a senior biological sciences mentor. Mentors are upperclassman biological sciences majors selected by the department; they have a tutoring background and have excelled in introductory biological sciences courses. The mentors provide increased academic support and serve as students' main guide to campus resources and opportunities. Additionally, LC students participate in a weekly 50-minute seminar led by a LC mentor. Seminar topics are generally academic in nature and focus particularly on study skills, metacognition, and research experience. Also discussed are general first-year issues, such as how best to communicate with professors and TAs and how to manage fast-paced coursework in a quarter system.

We note that patterns of co-enrollment differed across years. In Year 1, non-LC students were allowed to co-enroll in the same introductory biology section with LC students, while in Year 2 they were not. Likewise, in Year 1, non-LC students could co-enroll in the same freshman seminar sections along with the LC students, whereas in Year 2, they could not. These differences across years likely help to explain the stronger size and segmentation effects of the LC in Year 2 as seen in Figures 1 and 3.

Survey Instruments and Data Collection Design

Electronic surveys were sent to the entire freshman cohort during the final week of Fall term to collect data on friendship ties within the major, for both intervention participants and non-participants, and various aspects of student background to serve as controls. Information on LC participation, as well as additional student demographic data was provided by the university. These surveys took roughly 20 minutes to complete and were administered to all first-year students enrolled as biological sciences majors. Student completion of the fall surveys was tied to course credit, helping generate a response rate of over 93% (see Figure S6).

There were some differences in survey design across the two years. In Year 1, the campus learning management system's survey feature was used to administer the survey. This relatively unsophisticated survey tool required all questions to appear on the same page, and was unable to make use of display logic, text piping, or skip logic. As such, the question used to generate each friend's name was immediately followed by questions about that friend (i.e., name interpreter question). That is, students

were asked to list their first friend, and then immediately answer one to three follow-up questions about that friend, before moving on to list the next friend, answer follow-up questions about the second friend, and so on. In Year 2, however, Qualtrics was used to administer the survey. With the benefit of display logic and text piping, this survey asked students to first list up to 10 friends, before then moving to subsequent pages in which follow-up questions were asked about those friends.

In addition, the wording and nomination limit varied slightly across cohorts. In Year 1, we stated, “These questions ask you about friends who are also Bio Sci majors. We define a friend as someone who you enjoy spending time with.” In Year 2, we simply stated “Please list people in the Bio Sci major that you consider your friends.” And, we limited the number of friendship nominations to eight in the first year but set the limit at ten for the second year. Supplementary analyses show less than 1% of students named the max number in Year 1, and around 1% named the max in Year 2.

These methodological differences are likely responsible for the observed difference in average number of friends across the two years as evident in Figures 1 and S6. In particular, the order of the Year 1 friendship questions could have had a “training effect” whereby respondents realized that naming a friend led to several follow-up questions. This can increase the burden of data collection and inhibit the elicitation of additional names [1-2] The design of the Year 2 survey captured friendship nominations (and hence network size) prior to students being asked follow-up, name interpreter questions. Hence, there is no possibility for training within the survey. As a methodological artifact, the difference in number of friends across years means that we cannot compare the two years but does not affect our tests of differences between LC and non-LC students *within* a given year because the methods were consistent across students within the year. To adjust for these differences, we use year fixed effects in the RD analysis and estimate ERGMs separately for each year.

Summary of Cohorts

Figure S6 provides a description of the biological sciences freshman cohorts, over the two years of this study in terms of its composition, academics, and networks. These cohorts represent all first-time (i.e., non-transfer), biological sciences freshman students, drawn from administrative records of those who have declared the relevant major and are thus on the departmental roster. Descriptive statistics highlight that across cohorts LC students are disproportionately URM, first-generation college, low income, and female, compared to their non-LC counterparts. Academically, we see that in Year 2, LC students entered with slightly lower SAT math scores compared to Year 1 LC students and ended the fall term with a lower average cumulative GPA. Finally, with respect to networks, we see that mean outdegree increased across cohorts which, as discussed, we attribute to methodological changes.

Sample and Missing Data

Our sample comes from the roster of students identified as a Biological Sciences major each year. Given our high response rate and ability to draw on administrative records, there was a minimal amount of missing data; most covariates were missing less than three percent. For the RD analyses, we utilized listwise deletion to create our analytic sample, resulting in a final pooled sample size of N=1,854. For the ERGMs, we retained all students (Year 1 N=879; Year 2 N=1,083) and used mean imputation for missing covariate data since the ability to keep all cases is critical in network analysis to avoid losing important structural features. Outgoing friendship nominations of non-respondents were specified as missing, which allows them to be imputed during model fitting, but treats them as non-informative to parameter estimates.

Section 2: Description of RD Measures

Dependent Variables

There are many potential ways to conceptualize friendship ties, but we draw upon measures of the size, strength, structure, and composition of friendship networks since these dimensions index important aspects of resources and support [3]. To construct these outcomes, each respondent's friend nominations were matched to data from other respondents and administrative data provided by the university. All measures are constructed at the individual level from complete network (or sociocentric) data gathered at the major cohort level [4-5]. Additionally, our measures are limited to friends who are directly connected to a focal student (i.e., local neighborhood with distance of one).

Network size refers to the number of friends with whom a focal student is connected. In this analysis, we measure the number of ties in terms of total degree, where we take the total number of peers a focal student is connected to, without differentiating who named who as a friend (the focal student or the peer). Figure S7 presents a visualization of variation in network size, where three different networks are displayed; the upper left student is an isolate having no friends, the upper right focal student has eight friends, and the lower left focal student has three friends.

Tie strength reflects the idea that relationships vary along dimensions such as closeness, intensity, and meaningfulness. One indicator of strength in a dyad is whether it is significant enough that both members acknowledge the relationship by naming each other as friends. Such relationships are defined as mutual (or reciprocal). To capture how student networks vary in terms of overall strength, we calculate the count of all a students' ties that are reciprocated. Alternative analyses utilizing **1)** the *count of nonreciprocal* ties, **2)** the *proportion of reciprocal* ties, or **3)** the *count of persistent (or stable)* ties from week 2 to week 10 as the outcome, produce substantively similar results. Figure S8 provides a sociogram of two different friendship networks, where the focal student on the left has four mutual ties (i.e., four edges with arrows going in both directions), whereas the focal student on the right has four non-mutual ties (i.e., two incoming arrows and two outgoing arrows).

Network structure encapsulates a more holistic view of friendships by recognizing that students not only have friends, but that those friends may be connected to one another. When one's friends are themselves friends, then a group exists. Such social closure allows for the development of group norms, enhanced social influence, and greater exchange of resources. We use density as our measure of network structure, calculated as the number of observed ties among a respondent's named friends divided by the number of potential ties. In Figure S9, we present three different friendship network structures, where each focal student has the same number of friends, but they differ in the number of ties present among their friends.

Finally, network composition refers to the makeup of one's network. Although many characteristics could be considered, given the design of the LC, we expect friendships with LC participants to vary greatly depending upon whether a student belongs to the LC. Thus, we examine the proportion of friends in the learning community. Figure S10 provides a visualization of variation in network composition. If we imagine that the colors corresponded with LC participation, then the upper left network would indicate that all friends of the focal student are either LC participants or non-participants, whereas the other two networks have varying degrees of heterogeneity in terms of LC participation.

Control Variables

We account for differences in observed background characteristics by including covariates gathered from administrative and survey data to improve the precision of our estimates. Specifically, we include measures of gender (reference=female), first-generation student status (yes=1), low-income status (yes=1), Fall term cumulative GPA, high school GPA, SAT reading score, and SAT writing score provided through administrative sources. We also include a measure of race/ethnicity (reference=White) that was provided by individual students through administrative sources in Year 1 and primarily through survey data collection in Year 2, with imputation from administrative sources when survey information was missing. For this reason, four categories were used for the Year 1 cohort (i.e., White, Black/African American, Hispanic/Latinx, Asian/Asian American), whereas an additional "other" category was included for the Year 2 cohort that corresponded to students who identified as multiple racial/ethnic categories, or who checked the "other" category in the survey. Finally, we include a variable indicating survey completion status (completed=1), since network measures can be sensitive to missing data.

Section 3: Checking Conditions of RD

As a crucial first step to ensure an accurate estimate of the impact of the program through an RD framework, we must evaluate the main conditions of the model. As directed by Jacob et al. [6], we use a variety of graphical plots to explore the relationship between the rating variable and other measures of interest. Specifically, through these graphical plots and significance tests where applicable, we aim to **1)** examine whether the RD design should be considered "sharp" or "fuzzy", **2)** assess the internal validity of the RD design by examining potential manipulation of the running variable or differences in baseline characteristics at the cutoff, and **3)** visualize the discontinuity of the outcome variables at the cutoff to help explore potential functional form issues.

First, we used the *rdbwselect* function in STATA to estimate the optimal bandwidth on the pooled sample [7]. Although the optimal bandwidth varied across outcomes, we chose a bandwidth of ±70 points, since this closely matched similar previous studies [8]. Supplementary analyses using half and double the bandwidth size produced substantively similar results. As can be seen in Figures S11 and S12, we find evidence of a discontinuity in the probability of receiving the treatment at the cutoff. However, this discontinuity is not perfect since there is evidence of noncompliance, indicating that we should pursue a fuzzy RD design [9]. Next, we plot the density of the running variable using STATA's user-written *rddensity* command [10]. Significance tests of a discontinuity at the cutoff indicate that there is a significant jump for Year 2 ($p<.001$) but not Year 1 ($p>.10$)(see Figures S13 and S14). Finally, we plot the baseline characteristics of the students as a function of the assignment variable (see Figures S15 and S16). In general, we do not see much evidence of a discontinuity in baseline characteristics for Year 2, but there do seem to be some jumps for Year 1. Supplementary analyses reveal substantively similar effects across years. All RD models control for observed background characteristics to improve precision.

Section 4: Description of ERGMs and Estimated Effects

ERGM Overview

Whereas the RD analysis demonstrates the causal impact of the LC on friendship networks, it does not offer insight to *how* the LC had its demonstrated effect. Toward that end, the social network analysis was performed with the goal of discerning whether the impact of LC participation on friendship was directly due to LC organizational factors (i.e., course scheduling) versus alternative mechanisms that may have resulted from the assignment of students to courses. The alternative mechanisms we investigate reflect different ways that the students assigned to the LC could have reasonably been different from the students who did not participate in the LC. The general logic of our analysis was to **1)** estimate a base

ERGM that only included the hypothesized main effect of LC participation, **2)** estimate subsequent models that introduced effects to account for alternative friend selection mechanisms, and **3)** a full model that included foci effects as well as alternative factors simultaneously. ERGMs were estimated using the *statnet* package in R.

Because of scaling issues that arise with nonlinear outcomes, we converted estimated ERGM coefficients to marginal effects to compare models. To determine if an alternative mechanism is responsible for the observed effect of LC participation, we calculated the marginal effect of the respective LC effect in the base model and compared it to subsequent models that introduced potential confounds or mediators. If a confound or mediator were responsible for the observed effect of the LC on friendship, then the marginal effect of the LC would decrease compared to the base model. We also calculated how much of the main LC effect was explained (or mediated) by effects introduced to the model, following the method proposed by Duxbury [11] and implemented in the *ergMargins* package in R.

The ERGM itself is a multivariate network model that estimates the presence, versus absence, of friendships (or “ties”) conditional upon effects included in the model. The set of effects included in an ERGM capture particular configurations of ties in the network. All models included an *edges* effect, to control for the overall probability of observing a tie. Additional effects can incorporate individual, dyadic, or network properties as discussed below.

Unlike the RD analysis, the ERGM considered the entire major cohort for each year, with students designated as either LC participants or non-participants. Given our quasi-experimental design, with LC status exogenously assigned to students, we focus on distinguishing the total effect of LC assignment through the assignment of students to cohorts and sections from potential effects due to the manipulation of other population features. The alternative explanations we test are all based on individual and dyadic properties; hence, our specification is also referred to as a dyad-independence model [12].

ERGM Specification of Hypothesized Effects

We estimate a sequence of ERGMs to test each of the two hypotheses supported by the RD design. Each sequence of models begins with a base model that represents the main effect of LC participation on friendship. First, we consider the finding that LC participants had more friends than non-participants. The base model testing this effect uses a *nodecov* effect associated with student LC participation (see ref. 13 for a fuller description of ERGM effects). This effect calculates the sum of the LC participation covariate across the two students in each dyad. This sum evaluates to “0 if neither student is an LC participant, “1” if one student is an LC participant, and “2” if both are LC participants. The associated parameter estimate can be interpreted as the difference in the log-odds of observing a tie for a one-unit increase in this sum (e.g., for a dyad with no LC members to a dyad with one LC member). The results for the base model are reported as M1 in Figures S17a and S17b. As a robustness test, we estimated an alternate set of models that only considered friendships from the perspective of the student naming a friend (i.e., using the *nodeocov* effect). These models produced substantively similar findings (see Figures S19a and S19b).

The second series of models evaluates the finding that LC participants were more likely to name friends in the LC than were non-LC participants. This finding is tested with a dyadic effect capturing the combination of student LC participation in each dyad. The *nodemix* effect creates a separate dyadic indicator for all but one combination of LC participation status. Our specification created effects to represent friendships from one LC-participant to another LC-participant (LC → LC), a non-participant to a non-participant (non-LC → non-LC), and an LC-participant to a non-participant (LC → non-LC). This left friendships where a non-participant named an LC-participant as a friend as the reference category (non-

LC → LC). Our interest is in whether LC → LC dyads were more likely to exhibit friendships than non-LC → LC dyads (the reference category). Hence, we treat the LC → LC dyad parameter estimate as our indicator of segmentation. Results for this base model are reported as M1 in Figures S17a and S17b.

ERGM Specification of Alternatives

LC assignment was based on math SAT scores, which are correlated with student background characteristics. Hence, the composition of the LC was different from the overall composition of the major. (see Figure S6). One possibility is that the students assigned to the LC were more sociable or more likely to make friends due to background characteristics, such as race and gender [14]. Hence, we consider several factors that were associated with either LC placement or friendship volume, including race/ethnicity, gender, first-generation student status, low-income status, high school GPA, and commuter status. These attributes were entered into the model using *nodecov* effects or, for categorical attributes (i.e., race), a *nodefactor* effect (which specified a separate effect for each level of the factor, excluding one). Full model results are shown as M2 in Figures S17a, S17b, S18a, and S18b.

Another explanation for our observed findings is that the composition of the LC friendship pool affected the capacity for students to find friends who were similar to themselves. Homophily is one of the most common patterns found in human social relationships [15], including among university students [14]. Foci such as the LC have the potential to act as a filter, bringing together a set of individuals that is more homogenous than the broader population and promoting relationships among them [16-17]. If this were to occur, then LC students from backgrounds that are over-represented in the LC would have an easier time finding similar peers to befriend than students outside the LC. We test for this possibility using **1)** a set of effects that represent similarity on background factors within each dyad – *nodematch* effects for dichotomous measures and categorical measures (0=matching scores, 1=different scores), *absdiff* effects for continuous measures (reflecting the absolute difference between two students' scores) – and **2)** a set of interactions between similarity and whether both members of a dyad were LC participants ("1"=both LC participants, "0"=at least one student not an LC participant; created as dyadic measures and entered as *edgecov* effects). With this specification, the main effects of similarity capture the strength of homophily for dyads that did not include two LC participants, and the interactions represent how homophily among LC participants deviated from this main effect. Results are presented as M3 in Figures S17a, S17b, S18a, and S18b.

A third possibility is that the LC brought together students who were more likely to have a pre-existing friendship. For instance, LC participants may have been more likely to draw from the same high schools. For Year 2 only, we have information on which high school each student in our sample attended. We use this data to construct a dyadic covariate representing, for each pair of students in the sample, whether they attended the same high school (1=yes, 0=no). We included this covariate in the model with an *edgecov* term. Results are presented as M4 in Figures S17b and S18b.

It is possible that the aforementioned alternative explanations each accounted for a small part of the LC effect observed, but not enough to fully explain it away. Hence, we estimated a composite model that included all of the alternative explanations tested in M2-M4. We present this as M5 in Figures S17a, S17b, S18a, and S18b.

Our final models test the hypothesis that the effect of LC participation on friendships operated through assignment to the same classes and LC section (M6 in Figures S17a, S17b, S18a, and S18b). We focus on the three courses that all majors were required to take and were subject to the block-scheduling design: "Introduction to Biology", "Introduction to Chemistry", and "Freshman Seminar." For each of these

courses, we coded each student dyad “1” if they took the class together, and “0” if they did not. Similarly, dyads were coded “1” if both members were in the same LC section and “0” otherwise (including dyads that included LC non-participants). We used *edgecov* effects to test how these four foci affected friendships. M6 introduces these four effects along with the main LC effect of interest.

Average Marginal Effect (AME) Results

To evaluate the potential confounds represented by the alternative selection mechanisms, as well as the mediating effect of shared foci, we converted ERGM parameter estimates into partial average marginal effects following Duxbury [11]. Partial AMEs represent the direct effect of the LC predictor net of the other effects introduced to each model. Partial AMEs and 95% confidence intervals for each model are presented in Figure S20. Figure S21 presents these partial marginal effects and standard errors, along with the total AME, indirect AME of the LC effects on friendship (via the effects introduced to the model) and standard errors. We also calculate the percent of the total AME that is mediated by the partial AME.

SI Figures

Figure S1. T-test of Means on Network Outcomes by LC Status and Year

Network Outcome	Year	LC Participant (yes=1)	Mean	SD	P-value
Total Degree					
	1	1	2.94	2.08	
	1	0	2.50	1.98	0.005
	2	1	5.21	3.12	
	2	0	4.06	2.83	<0.001
Count of Mutual Ties					
	1	1	1.03	1.08	
	1	0	0.97	1.06	0.47
	2	1	2.06	1.61	
	2	0	1.73	1.42	0.002
Density					
	1	1	0.27	0.31	
	1	0	0.22	0.27	0.04
	2	1	0.25	0.23	
	2	0	0.25	0.24	0.86
Proportion of Friends in LC					
	1	1	0.76	0.29	
	1	0	0.12	0.25	<0.001
	2	1	0.82	0.23	
	2	0	0.08	0.18	<0.001

Figure S2. Logistic Regression of LC Participation on Odds of Isolation within Major Cohort

	M1 (Full sample)	M2 (Within bandwidth)
Learning Community Participant (yes=1)	0.45**	0.49**
	(.104)	(.133)
Individual-level Controls	X	X
Year Fixed Effects	X	X
N	1,854	1,031

†p<.10; *p<.05; **p<.01;***p<.001. Standard errors in parentheses.

NOTES: Results reported as odds ratios.

Models examine isolation as measured at the end of fall term among freshmen.

Covariates include: race/ethnicity (reference=White), gender, first-generation student status, low-income status, Fall term cumulative GPA, high school GPA, SAT reading, SAT writing, and survey completion status.

Figure S3. LATE Estimates from Pooled Local Polynomial Regression on Various Network Outcomes, LC Effect: Bandwidth ±70

Network Outcome Measure	M1	M2	M3	M4
Network Size				
Total degree	1.01†			
	(.559)			
Tie Strength				
Count of Reciprocal Ties		0.32		
		(.318)		
Network Structure				
Density			-0.09	
			(.066)	
Network Composition				
Proportion of Friends in LC				0.54***
				(.062)
Individual-level Controls	X	X	X	X
Year Fixed Effects	X	X	X	X
N	1,031	919	772	919

†p<.10; *p<.05; **p<.01;***p<.001. Robust standard errors in parentheses.

NOTES: Estimates adjust for fuzzy RD design.

Covariates adjusted include: race/ethnicity (reference=White), gender, first-generation student status, low-income status, Fall term cumulative GPA, high school GPA, SAT math score distance from cutoff, SAT math score distance squared, an interaction term between SAT math score distance from cutoff and whether the student was below the eligibility threshold, SAT reading, SAT writing, and survey completion status.

Model 1 includes all students; Model 2 is limited to students with at least one tie; Model 3 is limited to students with at least two ties; Model 4 is limited to students with at least one tie.

Figure S4. Predicted Network Outcomes Based on LATE Estimates

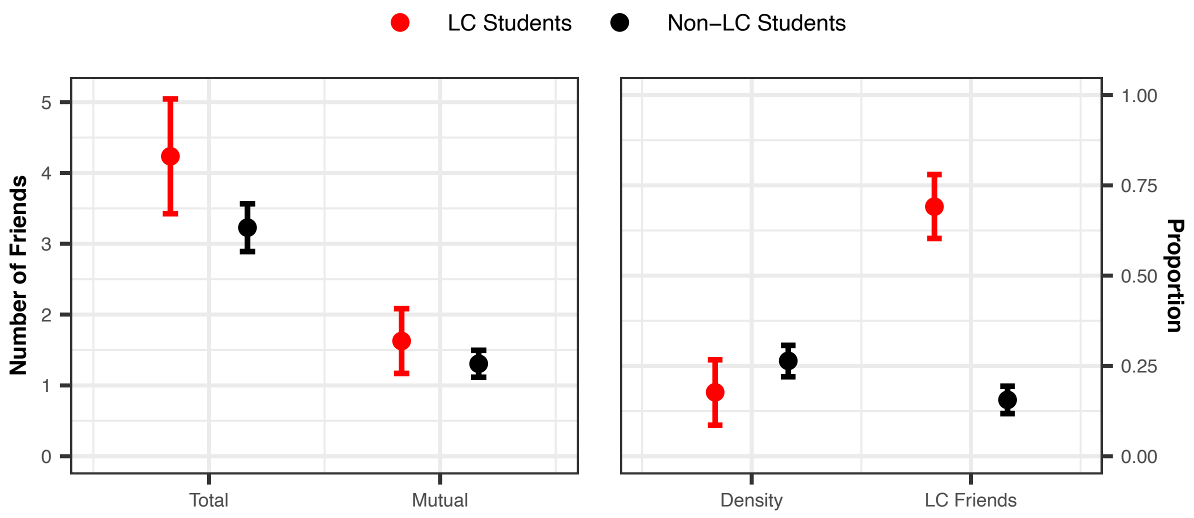

Figure S5. Learning Community Program Description

Year	Eligibility	Program Component				Attrition
		Additional Chem Prep	Peer Mentor	LC Seminar (1-hour weekly session)	LC/Non-LC Co-enrollment Practices	
1	<600 SAT Math	No	Yes; holds 1-hour weekly session	Yes; each quarter	Non-LC students allowed to co-enroll in LC intro bio section; Non-LC students allowed to co-enroll in freshman seminar sections	Bio: Students must earn a D- or above to move forward in the sequence. Chem: Students must earn a C- or above to move forward in the sequence.
2	Same as above	Chem 1X (fall)-Required[1]	Same as above	Same as above	Non-LC students not allowed to co-enroll in LC sections	Same as above

[1]Co-requisite with *Introduction to Chemistry* course so LC students remain on-sequence with the rest of the cohort in terms of Chemistry.

Figure S6. Descriptive Overview of Biological Sciences Freshman Cohorts

		Year 1			Year 2		
Composition		LC	Non-LC	Overall	LC	Non-LC	Overall
	Size of Cohort (N)	240	637	879	290	784	1083
	% URM	67.1	18.5	31.8	75.2	27.7	40.4
	% First-Generation College	77.4	40.5	50.7	75.0	43.1	51.7
	% Low-Income	51.3	22.8	30.6	60.1	31.0	38.8
	% Female	80.4	63.6	68.2	84.6	63.3	68.9
Academics							
	Mean SAT Math	516	656	617	498	658	616
	Mean SAT Reading	539	608	589	522	609	586
	Mean SAT Writing	514	606	581	502	607	579
	Mean Fall Term GPA	2.43	3.16	2.95	1.95	3.16	2.83
Networks							
	Mean Outdegree	1.88	1.66	1.72	3.42	2.75	2.93
	Mean Indegree	1.93	1.65	1.72	3.54	2.71	2.93
	Mean Total Degree	2.91	2.50	2.61	5.11	3.96	4.27
	Mean Count of Mutual Ties	1.02	0.96	0.98	1.96	1.67	1.75
	Mean Density	0.28	0.22	0.24	0.26	0.26	0.26
	Mean Proportion of Friends in LC	0.76	0.12	0.30	0.82	0.08	0.29
Survey							
	% Survey Completion	92.1	94.2	93.6	94.5	95.3	95.1

Figure S7. Examples of Variation in Network Size

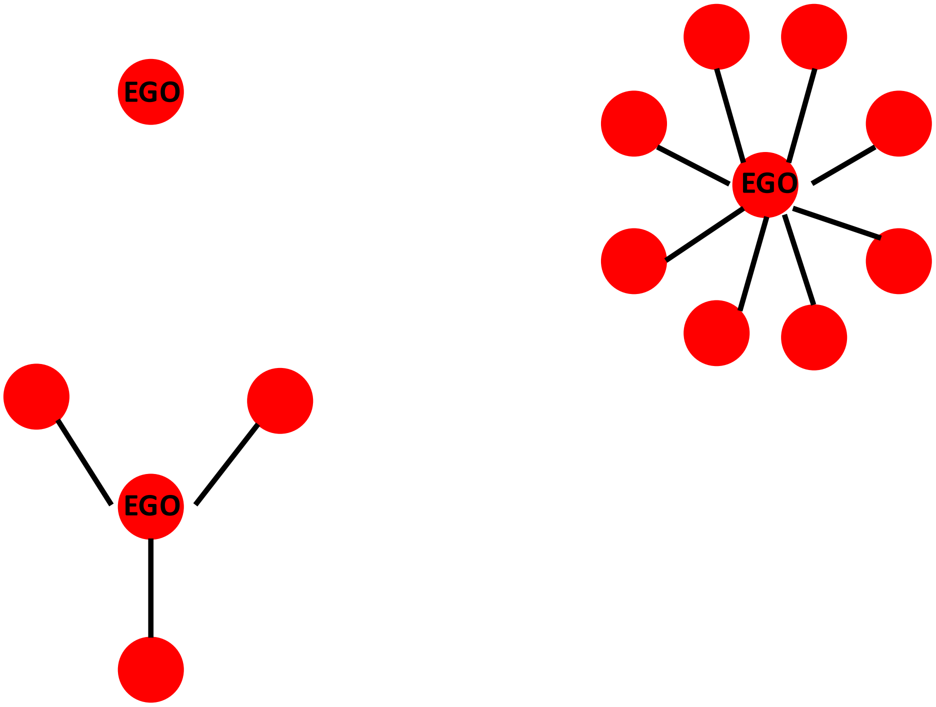

NOTE: Focal individual (ego) represented by middle node (or circle); alters represented by outer nodes.

Figure S8. Examples of Variation in Tie Strength

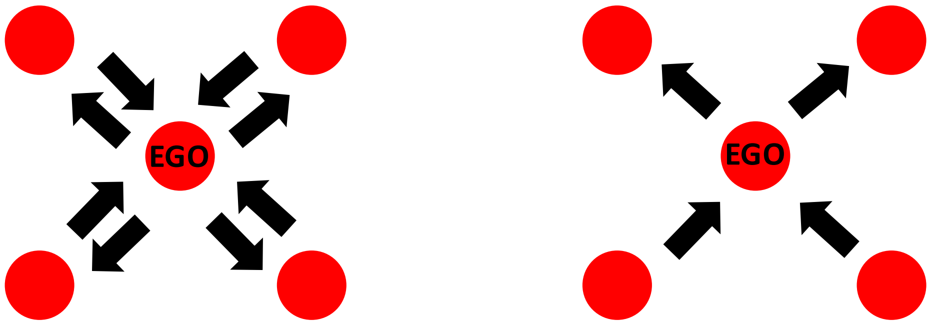

NOTE: Focal individual (ego) represented by middle node (or circle); alters represented by outer nodes; arrows indicate directionality of tie nomination.

Figure S9. Examples of Variation in Network Structure

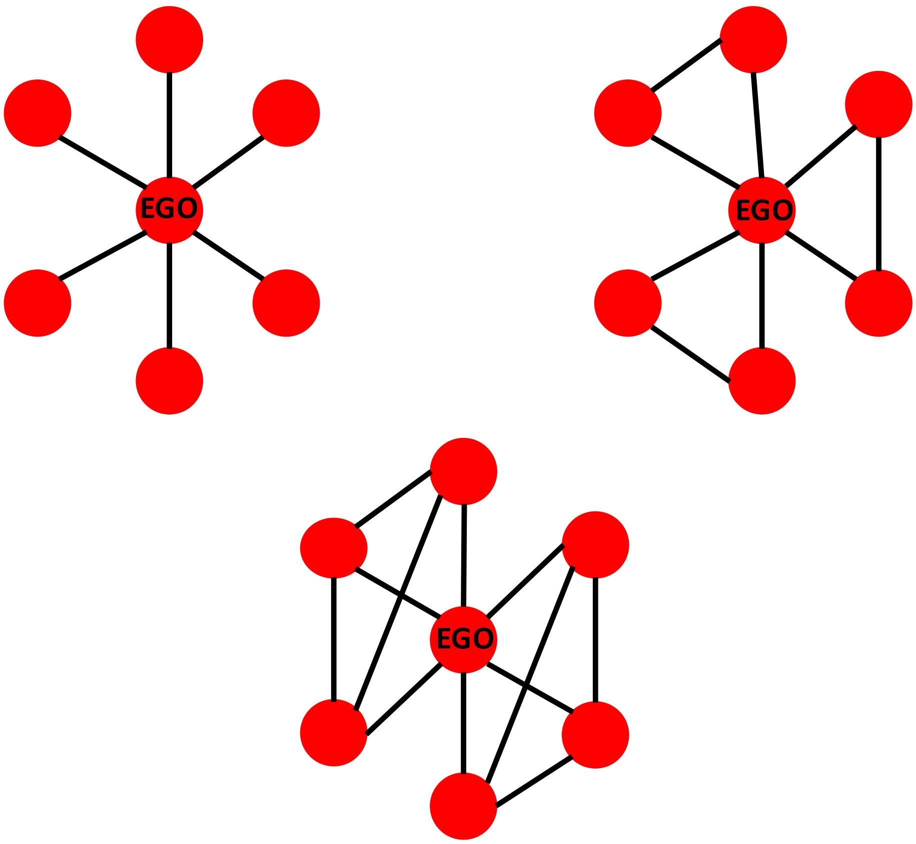

NOTE: Focal individual (ego) represented by middle node (or circle); alters represented by outer nodes.

Figure S10. Examples of Variation in Network Composition

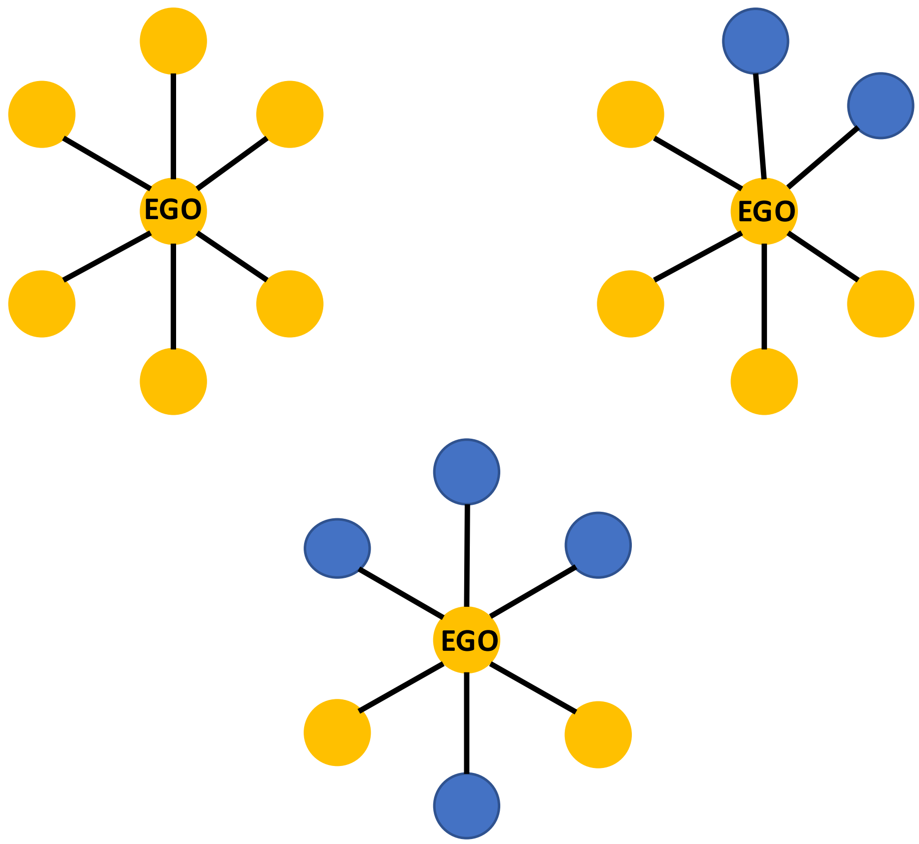

NOTE: Focal individual (ego) represented by middle node (or circle); alters represented by outer nodes; node color specifies group membership based on some characteristic, category, or other differentiating criteria.

Figure S11. Probability of Treatment by Distance from SAT Math Cutoff: Year 1

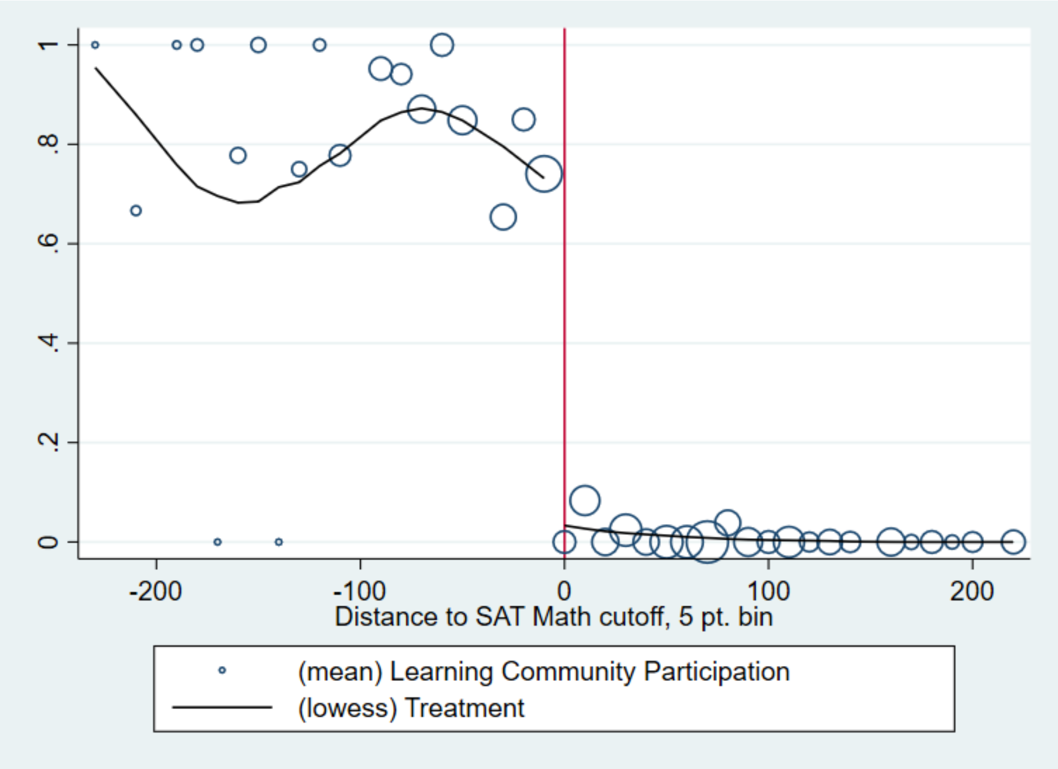

Figure S12. Probability of Treatment by Distance from SAT Math Cutoff: Year 2

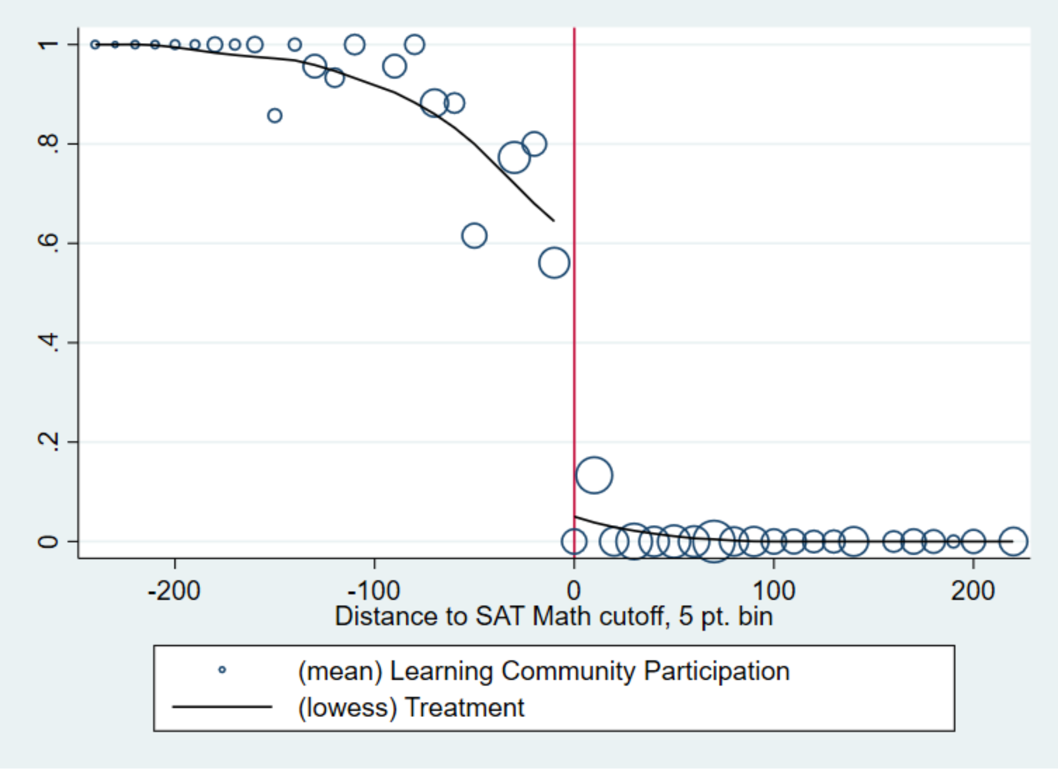

Figure S13. RD Manipulation Test using Local Polynomial Density Estimation: Year 1

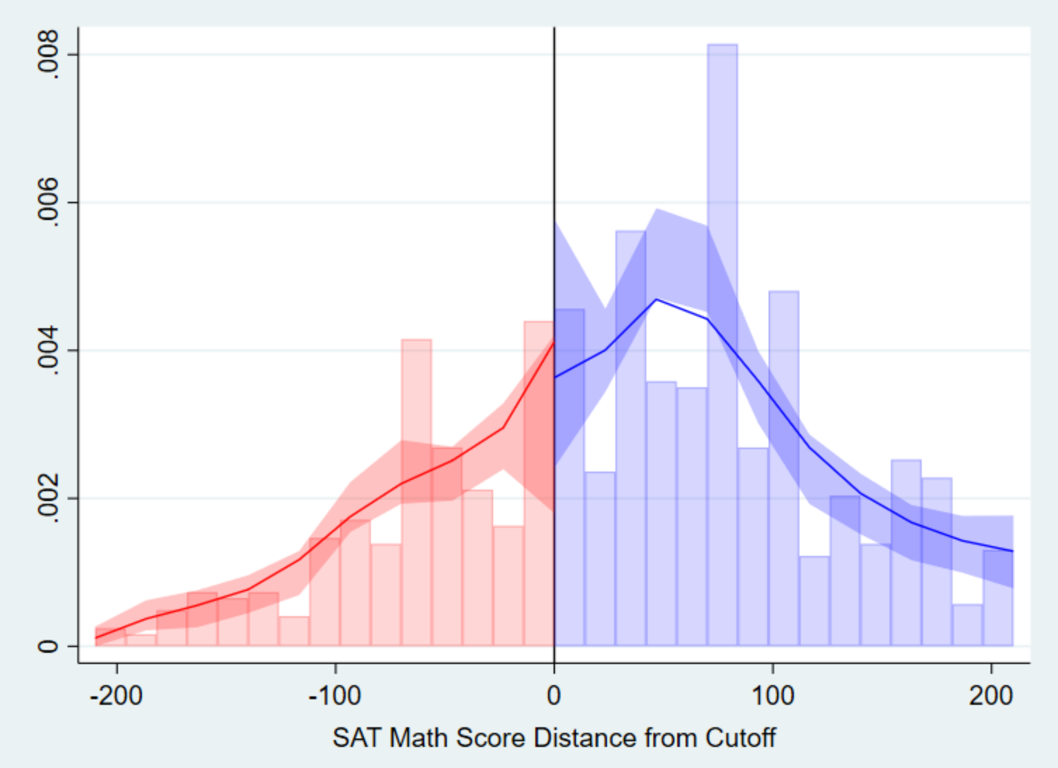

Figure S14. RD Manipulation Test using Local Polynomial Density Estimation: Year 2

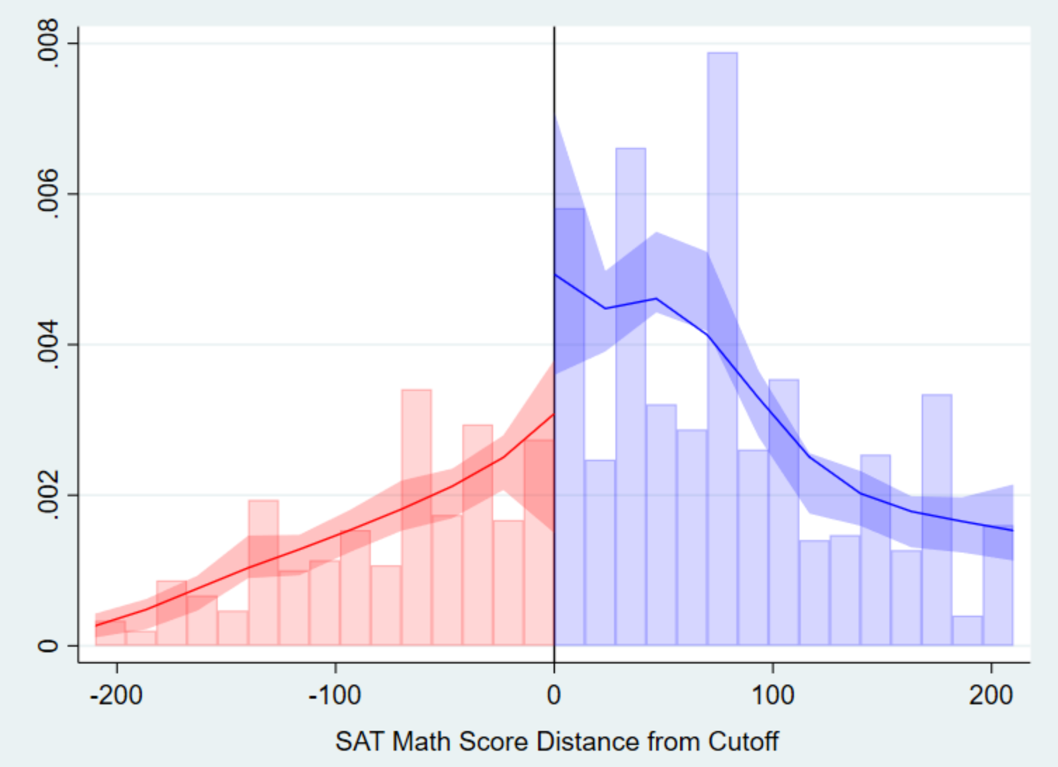

Figure S15. Distribution of Baseline Characteristics by Distance from SAT Math Cutoff: Year 1

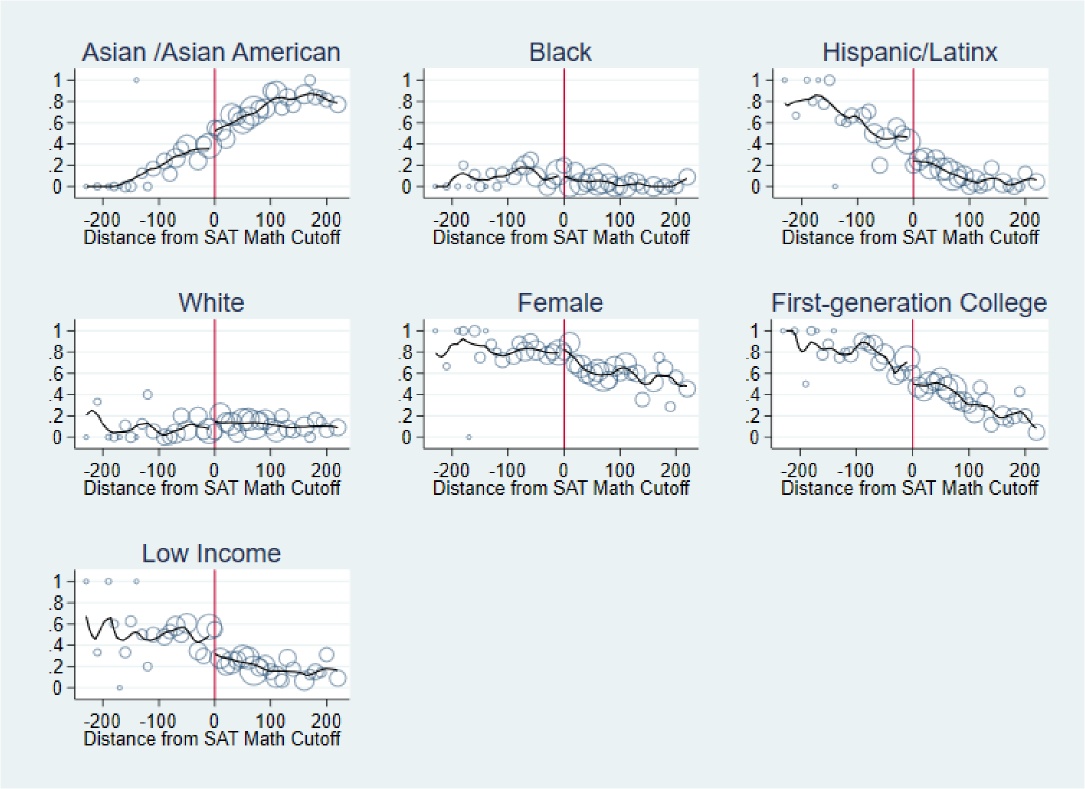

Figure S16. Distribution of Baseline Characteristics by Distance from SAT Math Cutoff: Year 2

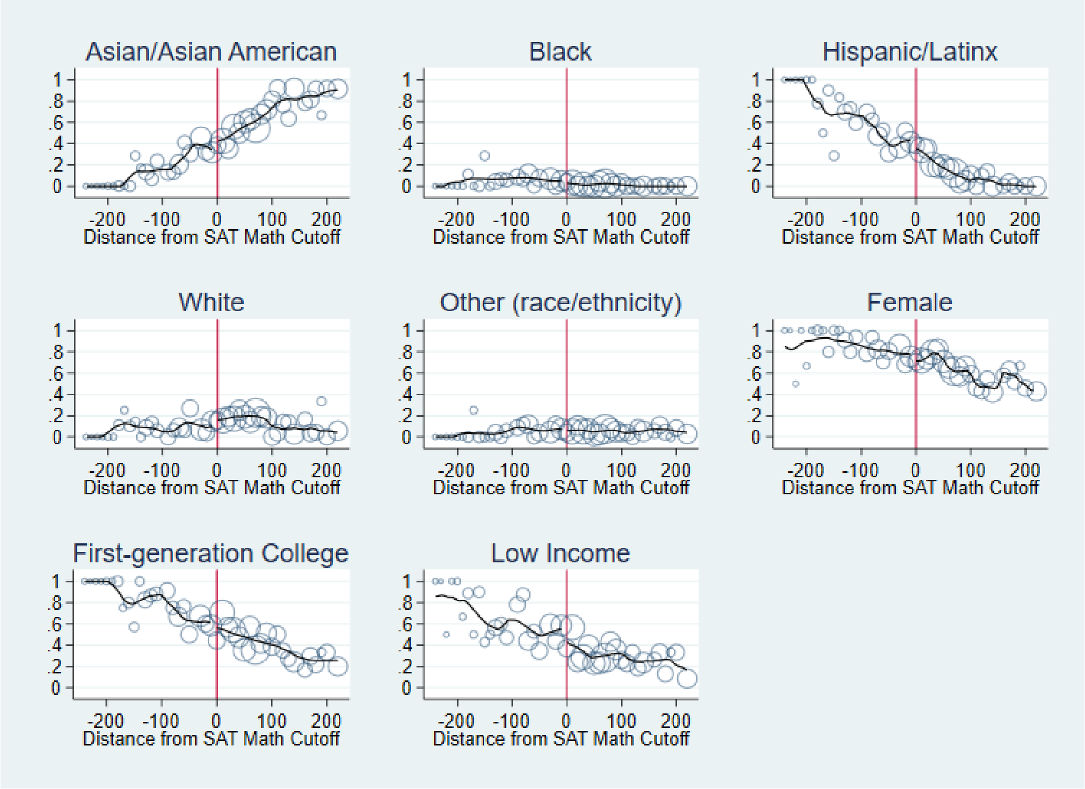

Figure S17a. Full ERGM results for models testing the effect of LC-participation on friendship volume (Year 1)

Year 1	M1			M2			M3			M4		M5			M6		
	coeff.	SE		coeff.	SE		coeff.	SE		coeff.	SE	coeff.	SE		coeff.	SE	
edges	-6.313	.035	***	-9.056	.838	***	-8.817	.157	***			-9.732	.967	***	-10.229	.052	***
nodecov																	
**LC participant (ref: non-participant)**	**.143**	**.040**	*******	**.222**	**.047**	*******	**.725**	**.089**	*******			**.707**	**.090**	*******	**-.482**	**.046**	*******
nodefactor																	
race (ref: Asian)																	
White				-.096	.061							.391	.059	***	.370		***
Latinx				-.204	.051	***						.030	.052		-.003		
Black				-.226	.082	***						.308	.081	***	.283		***
nodecov																	
first-generation (ref: non-first-generation)				-.059	.043							-.025	.044		-.051	.044	
female (ref: male)				.226	.042	***						-.034	.039		-.033	.039	
low-income (ref: not low-income)				.009	.045							.010	.045		-.006	.045	
high school GPA				.325	.102	***						.104	.115		.229	.116	*
commuter (ref: on-campus)				-.185	.047	***						-.198	.047	***	-.201	.047	***
nodematch																	
same race							.894	.064	***			1.096	.073	***	1.057	.073	***
same first generation							.207	.059	***			.183	.060	**	.167	.061	***
same gender							.889	.067	***			.908	.071	***	.916	.071	***
same LC participation status							1.703	.110	***			1.674	.112	***	.295	.126	**
absdiff																	
high school GPA							-.973	.188	***			-.956	.206	***	-.757	.209	***
edgecov																	
LC participant X same race							-.051	.129				-.110	.138		-.060	.138	
LC participant X same first generation							-.442	.130	***			-.339	.138	*	-.277	.138	*
LC participant X abs.diff high school GPA							1.093	.386	***			1.018	.387	**	.978	.391	**
LC participant X same gender							-.493	.143	***			-.489	.144	***	-.464	.145	***
same bio course															1.400	.059	***
same chem course															.947	.066	***
same freshman seminar															.392	.056	***
same LC section															2.245	.115	***
BIC	21941			21964			20884					20922			19418		

Figure S17b. Full ERGM results for models testing the effect of LC-participation on friendship volume (Year 2)

Year 2	M1		M2		M3		M4		M5		M6	
	coeff.	SE	coeff.	SE	coeff.	SE	coeff.	SE	coeff.	SE	coeff.	SE
edges	-5.968	.025 ***	-6.102	.553 ***	-8.491	.106 ***	-6.240	.027 ***	-9.425	.640 ***	-8.972	.654 ***
nodecov												
**LC participant (ref: non-participant)**	**.253**	**.027 *****	**.339**	**.033 *****	**.824**	**.059 *****	**.380**	**.027 *****	**.776**	**.060 *****	**-.284**	**.066 *****
nodefactor												
race (ref: Asian)												
White			-.077	.041 †					.360	.040 ***	.333	.040 ***
Latinx			-.076	.035 *					.141	.038 ***	.097	.037 ***
Black			-.042	.077					.494	.079 ***	.483	.080 ***
Other			.008	.055					.511	.056 ***	.521	.056 ***
nodecov												
first-generation (ref: non-first-generation)			-.003	.030					.020	.030	-.001	.031
female (ref: male)			-.142	.028 ***					-.282	.027 ***	-.293	.027 ***
low-income (ref: not low-income)			-.118	.029 ***					-.136	.030 ***	-.131	.030 ***
high school GPA			.065	.067					.149	.076 .	.225	.078 ***
commuter (ref: on-campus)			-.230	.033 ***					-.348	.032 ***	-.337	.032 ***
nodematch												
same race					.765	.043 ***			.872	.050 ***	.873	.051 ***
same first generation					.264	.042 ***			.158	.043 ***	.149	.043 ***
same gender					.513	.044 ***			.628	.045 ***	.625	.046 ***
same LC participation status					1.972	.078 ***			1.733	.079 ***	-.350	.100 ***
absdiff												
high school GPA					-.160	.128			-.066	.139	.086	.141
edgecov												
LC participant X same race					-.219	.085 ***			-.240	.096 *	-.154	.096
LC participant X same first generation					-.301	.088 ***			-.233	.093 *	-.175	.094 †
LC participant X abs.diff high school GPA					-.083	.219			.105	.222	-.039	.224
LC participant X same gender					-.283	.096 ***			-.083	.099	-.054	.101
same high school							4.274	.051 ***	3.980	.054 ***	4.055	.056 ***
same bio course											1.471	.048 ***
same chem course											.698	.048 ***
same freshman seminar											.387	.047 ***
same LC section											2.464	.073 ***
BIC	43261		43282		41095		39863		37905		35305	

Figure S18a. Full ERGM results for models testing the effect of LC-participation on friendship segmentation (Year 1)

Year 1	M1		M2		M3		M4		M5		M6	
	coeff.	SE	coeff.	SE	coeff.	SE	coeff.	SE	coeff.	SE	coeff.	SE
edges	-7.180	.092 ***	-9.849	.843 ***	-8.040	.120 ***			-8.972	.961 ***	-10.658	.970 ***
nodemix												
LC status (reference: non-LC → LC)												
non-LC → non-LC	1.119	.098 ***	1.040	.101 ***	.926	.099 ***			.914	.102 ***	.724	.106 ***
LC → non-LC	-.108	.134	-.108	.134	-.108	.134			-.108	.134	-.108	.135
**LC → LC**	**2.076**	**.107 *****	**2.155**	**.110 *****	**2.375**	**.195 *****			**2.328**	**.197 *****	**-.240**	**.220**
nodefactor												
race (ref: Asian)												
White			-.096	.061					.391	.059 ***	.370	.060 ***
Latinx			-.205	.051 ***					.030	.052	-.003	.051
Black			-.226	.082 ***					.308	.081 ***	.283	.082 ***
nodecov												
first-generation (ref: non-first-generation)			-.059	.043					-.025	.044	-.051	.044
female (ref: male)			.226	.042 ***					-.034	.039	-.033	.039
low-income (ref: not low-income)			.009	.045					.010	.045	-.006	.045
high school GPA			.326	.102 ***					.104	.115	.229	.116 *
commuter (ref: on-campus)			-.185	.047 ***					-.198	.047 ***	-.201	.047 ***
nodematch												
same race					.894	.064 ***			1.096	.073 ***	1.057	.073 ***
same first generation					.207	.059 ***			.183	.060 **	.167	.061 ***
same gender					.889	.067 ***			.908	.071 ***	.916	.071 ***
absdiff												
high school GPA					-.973	.188 ***			-.956	.206 ***	-.757	.209 ***
edgecov												
LC participant X same race					-.051	.129			-.110	.138	-.060	.138
LC participant X same first generation					-.442	.130 ***			-.339	.138 *	-.277	.138 *
LC participant X abs.diff high school GPA					1.093	.386 ***			1.018	.387 **	.978	.391 **
LC participant X same gender					-.493	.143 ***			-.489	.144 ***	-.464	.145 ***
same bio course											1.400	.059 ***
same chem course											.947	.066 ***
same freshman seminar											.392	.056 ***
same LC section											2.245	.115 ***
BIC	21321		21344		20896				20935		19419	

Figure S18b. Full ERGM results for models testing the effect of LC-participation on friendship segmentation (Year 2)

Year 2	M1		M2		M3		M4		M5		M6	
	coeff.	SE	coeff.	SE	coeff.	SE	coeff.	SE	coeff.	SE	coeff.	SE
edges	-6.920	.069 ***	-6.971	.555 ***	-7.591	.086 ***	-6.978	.069 ***	-8.576	.636 ***	-9.183	.651 ***
nodemix												
LC status (reference: non-LC → LC)												
non-LC → non-LC	1.243	.073 ***	1.158	.075 ***	1.072	.074 ***	1.056	.073 ***	.883	.077 ***	-.140	.087
LC → non-LC	-.153	.102	-.150	.102	-.159	.102	-.152	.102	-.155	.103	-.156	.103
**LC → LC**	**2.401**	**.078 *****	**2.489**	**.080 *****	**2.721**	**.134 *****	**2.343**	**.078 *****	**2.435**	**.135 *****	**-.709**	**.160 *****
nodefactor												
race (ref: Asian)												
White			-.077	.041 †					.360	.040 ***	.334	.040 ***
Latinx			-.076	.035 *					.142	.038 ***	.097	.037 ***
Black			-.042	.077					.494	.079 ***	.483	.080 ***
Other			.008	.055					.511	.056 ***	.521	.056 ***
nodecov												
first-generation (ref: non-first-generation)			-.003	.030					.020	.030	-.001	.031
female (ref: male)			-.142	.028 ***					-.282	.027 ***	-.293	.027 ***
low-income (ref: not low-income)			-.118	.029 ***					-.136	.030 ***	-.131	.030 ***
high school GPA			.065	.068					.149	.076 .	.225	.078 ***
commuter (ref: on-campus)			-.231	.033 ***					-.348	.032 ***	-.337	.032 ***
nodematch												
same race					.765	.043 ***			.873	.050 ***	.874	.051 ***
same first generation					.264	.042 ***			.158	.043 ***	.149	.043 ***
same gender					.513	.044 ***			.628	.045 ***	.625	.046 ***
absdiff												
high school GPA					-.161	.128			-.066	.139	.085	.141
edgecov												
LC participant X same race					-.219	.085 ***			-.241	.096 *	-.154	.096
LC participant X same first generation					-.301	.088 ***			-.233	.093 *	-.175	.094 †
LC participant X abs.diff high school GPA					-.083	.219			.105	.222	-.039	.224
LC participant X same gender					-.283	.096 ***			-.083	.099	-.054	.101
same high school							4.029	.051 ***	3.980	.054 ***	4.055	.056 ***
same bio course											1.471	.048 ***
same chem course											.698	.048 ***
same freshman seminar											.387	.047 ***
same LC section											2.464	.073 ***
BIC	41587		41609		41106		38468		37916		35614	

Figure S19a. Full ERGM results for models testing the effect of LC-participation on friendship volume (alternative specification of attribute effects - Year 1)

Year 1	M1		M2		M3		M4		M5		M6	
	coeff.	SE	coeff.	SE	coeff.	SE	coeff.	SE	coeff.	SE	coeff.	SE
edges	-6.266	.031 ***	-7.578	.591 ***	-8.149	.127 ***			-9.240	.679 ***	-9.639	.673 ***
nodeocov												
**LC participant (ref: non-participant)**	**.124**	**.056 ***	**.190**	**.066 *****	**.414**	**.114 *****			**.713**	**.090 *****	**-.394**	**.112 *****
nodeofactor												
race (ref: Asian)												
White			-.073	.086					.489	.094 ***	.472	.094 ***
Latinx			-.129	.072 †					.112	.081	.082	.080
Black			-.125	.112					.450	.120 ***	.429	.120 ***
nodeocov												
first-generation (ref: non-first-generation)			-.127	.061 *					-.101	.062	-.109	.063 †
female (ref: male)			.309	.060 ***					.048	.064	.059	.064
low-income (ref: not low-income)			-.025	.064					-.028	.064	-.039	.064
high school GPA			.300	.144 *					.080	.158	.199	.159
commuter (ref: on-campus)			-.156	.066 **					-.169	.066 *	-.178	.066 ***
nodematch												
same race					.812	.062 ***			1.049	.072 ***	1.067	.071 ***
same first generation					.154	.058 ***			.184	.060 **	.204	.060 ***
same gender					.795	.064 ***			.878	.070 ***	.937	.070 ***
same LC participation status					1.256	.099 ***			1.683	.111 ***	.615	.092 ***
absdiff												
high school GPA					-1.168	.188 ***			-.966	.197 ***	-.717	.194 ***
edgecov												
LC participant X same race					.201	.129			-.119	.136	-.209	.129
LC participant X same first generation					-.165	.132			-.345	.134 *	-.444	.126 ***
LC participant X abs.diff high school GPA					1.974	.358 ***			1.042	.386 **	.548	.371
LC participant X same gender					.025	.137			-.504	.144 ***	-.711	.124 ***
same bio course											1.373	.059 ***
same chem course											.903	.066 ***
same freshman seminar											.399	.056 ***
same LC section											2.135	.109 ***
BIC	21949		22005		20929				20945		19455	

Figure S19b. Full ERGM results for models testing the effect of LC-participation on friendship volume (alternative specification of attribute effects - Year 2)

Year 2	M1			M2			M3			M4			M5			M6		
	coeff.	SE		coeff.	SE		coeff.	SE		coeff.	SE		coeff.	SE		coeff.	SE	
edges	-5.889	.021	***	-5.661	.385	***	-7.817	.093	***	-6.123	.024	***	-8.504	.436	***	-7.829	.437	***
nodeocov																		
**LC participant (ref: non-participant)**	**.238**	**.038**	*******	**.267**	**.047**	*******	**.564**	**.084**	*******	**.382**	**.039**	*******	**.809**	**.060**	*******	**-.300**	**.083**	*******
nodeofactor																		
race (ref: Asian)																		
White				-.049	.058								.424	.063	***	.395	.063	***
Latinx				.013	.050								.257	.057	***	.228	.057	***
Black				-.033	.111								.486	.116	***	.479	.118	***
Other				.034	.079								.547	.085	***	.558	.086	***
nodeocov																		
first-generation (ref: non-first-generation)				-.030	.042								-.016	.044		-.038	.044	
female (ref: male)				-.030	.040								-.208	.043	***	-.207	.044	***
low-income (ref: not low-income)				-.106	.041	***							-.137	.042	**	-.125	.042	***
high school GPA				-.033	.094								.016	.101		.066	.103	
commuter (ref: on-campus)				-.124	.045	***							-.265	.046	***	-.250	.047	***
nodematch																		
same race							.688	.042	***				.768	.049	***	.788	.049	***
same first generation							.201	.041	***				.171	.043	***	.177	.043	***
same gender							.428	.042	***				.567	.046	***	.578	.046	***
same LC participation status							1.511	.076	***				1.811	.079	***	-.131	.080	
absdiff																		
high school GPA							-.374	.128	***				-.150	.133		-.005	.133	
edgecov																		
LC participant X same race							-.011	.086					-.271	.093	**	-.253	.091	***
LC participant X same first generation							.004	.089					-.262	.091	**	-.272	.087	***
LC participant X abs.diff high school GPA							.648	.209	***				.050	.221		-.228	.214	
LC participant X same gender							.311	.094	***				-.194	.099	*	-.265	.087	***
same high school										4.216	.050	***	3.920	.053	***	3.993	.055	***
same bio course																1.463	.048	***
same chem course																.675	.047	***
same freshman seminar																.378	.047	***
same LC section																2.423	.071	***
BIC	43308			43415			41220			39962			38151			36326		

Figure S20. Average Marginal Effects (AME) Corresponding to LC Size and Segmentation Effects in Figures S17 and S18

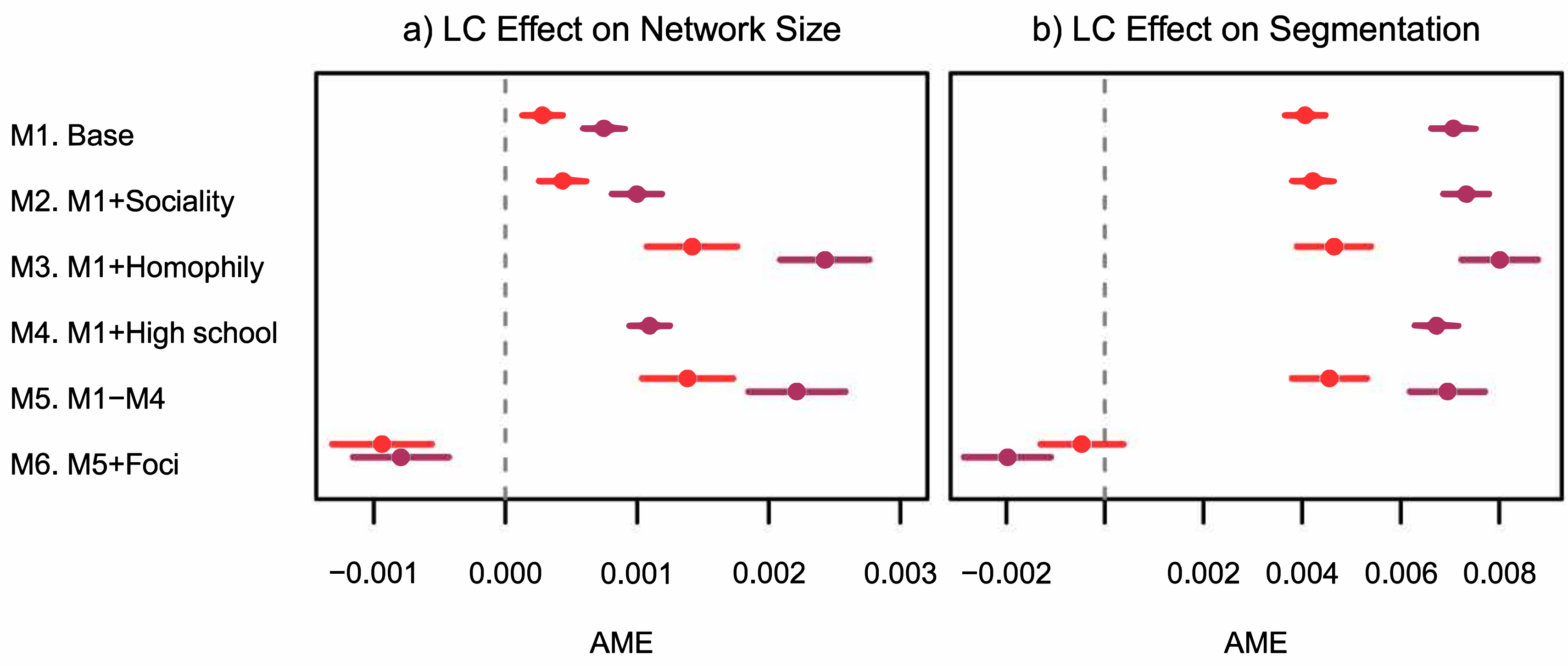

Figure S21. Mediation Test of LC Using Series of ERGMs

		Year 1						Year 2					
		Total AME	Partial AME	SE	Indirect AME	SE	Percent Mediated	Total AME	Partial AME	SE	Indirect AME	SE	Percent Mediated
Size													
M1a	Base	.00028	.00028	.00008				.00075	.00075	.00008			
M2a	Sociality	.00028	.00043	.00009	-.00015	.00010	-55.1	.00075	.00100	.00010	-.00025	.00010	-33.8
M3a	Homophily	.00028	.00142	.00017	-.00114	.00018	-405.8	.00075	.00243	.00017	-.00168	.00018	-224.9
M4a	Same HS	--	--	--	--	--	--	.00075	.00110	.00008	-.00035	.00011	-46.9
M5a	M1-M4	.00028	.00138	.00018	-.00110	.00018	-393.4	.00075	.00221	.00019	-.00147	.00019	-196.5
M6a	M5+Foci	.00028	-.00094	.00019	.00122	.00021	434.6	.00075	-.00079	.00019	.00154	.00020	206.3
Segmentation													
M1b	Base	.00407	.00407	.00021				.00707	.00707	.00023			
M2b	Sociality	.00407	.00422	.00021	-.00016	.00008	-3.8	.00707	.00733	.00024	-.00026	.00032	-3.7
M3b	Homophily	.00407	.00465	.00038	-.00058	.00028	-14.3	.00707	.00800	.00039	-.00094	.00045	-13.2
M4b	Same HS	--	--	--	--	--	--	.00707	.00672	.00022	.00034	.00032	4.9
M5b	M1-M4	.00407	.00455	.00038	-.00049	.00029	-12.0	.00707	.00695	.00039	.00012	.00045	1.7
M6b	M5+Foci	.00407	-.00047	.00043	.00453	.00054	111.5	.00707	-.00198	.00045	.00905	.00050	128.0

NOTES: Average Marginal Effect (AME).

Total AME is the average change in tie probability for a one-unit change in the predictor (LC status or LC dyad).

Partial AME is the direct effect of the LC predictor net of effects introduced to the model.

Indirect AME is the average change in tie probability that attributable to the LC indirectly through the confounding or mediating effect introduced.

The LC predictor is student LC status (participant vs. non-participant) in size models and LC -> LC dyad vs. a non-LC participant -> LC participant dyad in segmentation models.

SI References

1. Fischer CS (2009) The 2004 GSS finding of shrunken social networks: An artifact?. *Am Sociol Rev* 74(4):657-669.
2. Paik A, Sanchagrin K (2013) Social isolation in America: An artifact. *Am Sociol Rev* 78(3):339-360.
3. McCabe JM (2016) *Connecting in College: How Friendship Networks Matter for Academic and Social Success* (University of Chicago Press, Chicago, IL).
4. Crossley N, Bellotti E, Edwards G, Everett MG, Koskinen J, Tranmer M (2015) *Social Network Analysis for Ego-Nets: Social Network Analysis for Actor-Centred Networks* (Sage, Thousand Oaks, CA).
5. Perry BL, Pescosolido BA, Borgatti SP (2018) *Egocentric Network Analysis: Foundations, Methods, and Models* (Cambridge University Press, Cambridge, UK).
6. Jacob R, Zhu P, Somers MA, Bloom H (2012) *A Practical Guide to Regression Discontinuity* (MDRC, New York, NY).
7. Calonico S, Cattaneo MD, Farrell MH (2020) Optimal bandwidth choice for robust bias-corrected inference in regression discontinuity designs. *Econom J* 23(2):192-210.
8. Xu D, Solanki S, McPartlan P, Sato B (2018) EASEing students into college: The impact of multidimensional support for underprepared students. *Educ Res* 47(7):435-450.
9. Imbens GW, Lemieux T (2008) Regression discontinuity designs: A guide to practice. *J Econom* 142(2):615-635.
10. Cattaneo MD, Jansson M, Ma X (2018) Manipulation testing based on density discontinuity. *Stata J* 18(1):234-261.
11. Duxbury SW (2021) The Problem of Scaling in Exponential Random Graph Models. *Sociol Methods Res* DOI:10.1177/0049124120986178:1-39.
12. Robins G, Pattison P, Kalish Y, Lusher D (2007) An introduction to exponential random graph (p*) models for social networks. *Soc Networks* 29(2):173-191.
13. Morris M, Handcock MS, Hunter DR (2008) Specification of exponential-family random graph models: terms and computational aspects. *J Stat Softw* 24(4):1548-7660.
14. Lewis K, Kaufman J, Gonzalez M, Wimmer A, Christakis N (2008) Tastes, ties, and time: A new social network dataset using Facebook.com. *Soc Networks* 30(4):330-342.
15. McPherson M, Smith-Lovin L, Cook JM (2001) Birds of a feather: Homophily in social networks. *Annu Rev Sociol* 27(1):415-444.
16. Feld SL (1981) The focused organization of social ties. *AJS* 86(5):1015-1035.
17. Feld SL (1982) Social structural determinants of similarity among associates. *Am Sociol Rev* 47(6):797-801.
